# **Iron (II)-based metal-organic framework nanozyme for boosting tumor ferroptosis through inhibiting DNA damage repair and system Xc** ^**-**^

**DOI:** 10.1186/s12951-024-02508-2

**Published:** 2024-05-08

**Authors:** Panpan Xue, Huilan Zhuang, Tingjie Bai, Xuemei Zeng, Jinpeng Deng, Sijie Shao, Shuangqian Yan

**Affiliations:** 1 The Straits Institute of Flexible Electronics (SIFE, Future Technologies), The Straits Laboratory of Flexible Electronics (SLoFE), Fujian Normal University, Fuzhou, Fujian 350117 China; 2 Key Laboratory of Innate Immune Biology of Fujian Province, Biomedical Research Center of South China, College of Life Sciences, Fujian Normal University, 1 Keji Road, Fuzhou, 350117 PR China

**Keywords:** Metal organic framework, Disulfide bond, Autophagy, Ferroptosis

## Abstract

Development of ferroptosis-inducible nanoplatforms with high efficiency and specificity is highly needed and challenging in tumor ferrotherapy. Here, we demonstrate highly effective tumor ferrotherapy using iron (II)-based metal-organic framework (Fe*ss*MOF) nanoparticles, assembled from disulfide bonds and ferrous ions. The as-prepared Fe*ss*MOF nanoparticles exhibit peroxidase-like activity and pH/glutathione-dependent degradability, which enables tumor-responsive catalytic therapy and glutathione depletion by the thiol/disulfide exchange to suppress glutathione peroxidase 4, respectively. Upon PEGylation and Actinomycin D (ActD) loading, the resulting Fe*ss*MOF/ActD-PEG nanoplatform induces marked DNA damage and lipid peroxidation. Concurrently, we found that ActD can inhibit Xc^−^ system and elicit ferritinophagy, which further boosts the ferrotherapeutic efficacy of the Fe*ss*MOF/ActD-PEG. In vivo experiments demonstrate that our fabricated nanoplatform presents excellent biocompatibility and a high tumor inhibition rate of 91.89%.

**Supplementary Information:**

The online version contains supplementary material available at 10.1186/s12951-024-02508-2.

## Introduction

Breast cancer has been considered as the first most common cause of death in women [1–5]. Despite substantial advances in diagnostic techniques and combinatorial therapy, effective treatment of breast cancer with good prognosis and high survival remains a challenge, mainly due to the acquired resistance and invasive nature of malignant tumors [6, 7]. Therefore, an urgent need exists for the development of novel therapeutic approaches to breast tumor treatments.

It has been evidenced that ferroptosis is a powerful manner for tumor therapy, especially for breast tumors [8–12]. Ferroptosis is an emerging type of non-apoptotic regulated cell death by which to treat breast tumors [13–15]. The mechanism involved in ferroptosis has been ascribed to the excessive accumulation of lipid peroxidation (LPO) through the iron-dependent Fenton reaction [16]. Specifically, iron-mediated generation of reactive oxygen species (ROS) can peroxide membranous unsaturated fatty acids to induce cellular damage and ferroptotic death [10, 17]. Although ferroptosis can bypass apoptotic-associated tolerance and concert with apoptosis to enhance tumor treatments, tumoral antioxidants may debilitate ferroptosis by perishing the generated ROS and LPO [18, 19]. For instance, reductive glutathione (GSH) can remove ROS and serve as a cofactor for glutathione peroxidase 4 (GPX4) that inhibits ferroptosis [20, 21]. In this regard, the GPX4 has been considered a major ferroptotic suppression protein that can significantly detoxify lipid peroxidation [22, 23].

Small molecule-based ferroptosis inducers have been used for ferrotherapy by inhibiting cystine/glutamate antiporter protein (system Xc^−^) or GPX4 [24, 25]. However, most of those molecular inducers encounter dilemmas of poor water solubility and low tumor-targeting efficiency and the corresponding treatments are often accompanied by nephrotoxicity [26]. Nanoparticles can improve the targeting and biocompatibility of the ferroptosis inducers [27–29]. In addition, many kinds of nanoparticles hold enzyme-mimicking activities, which have been widely utilized in ferrotherapy [30]. For example, Fe_3_O_4_ nanoparticle has a peroxidase-like property that can catalyze intratumoral H_2_O_2_ to ^•^OH to induce cell damage and ferroptosis [31, 32]. Besides, GSH oxidase-mimic nanomaterials are capable trigger cellular ferroptotic death by GSH consumption [33]. Nevertheless, cell damage can be repaired by cellular inherent repair mechanisms [34]. And the GSH consumption will be recomposed by the continual cystine uptake through system Xc^−^ and following GSH synthesis in tumor cells [35, 36]. It must also be mentioned that ferric ion (Fe^3+^)-based nanoparticles have low Fenton activities compared to the ferrous ion (Fe^2+^)-contained materials [37]. Therefore, designing powerful nanoplatforms that is consisted of Fe^2+^ with properties of GSH oxidation, system Xc^−^ inhibition, and cell damage repair suppression are highly needed in tumor ferrotherapy.

**Scheme 1:**
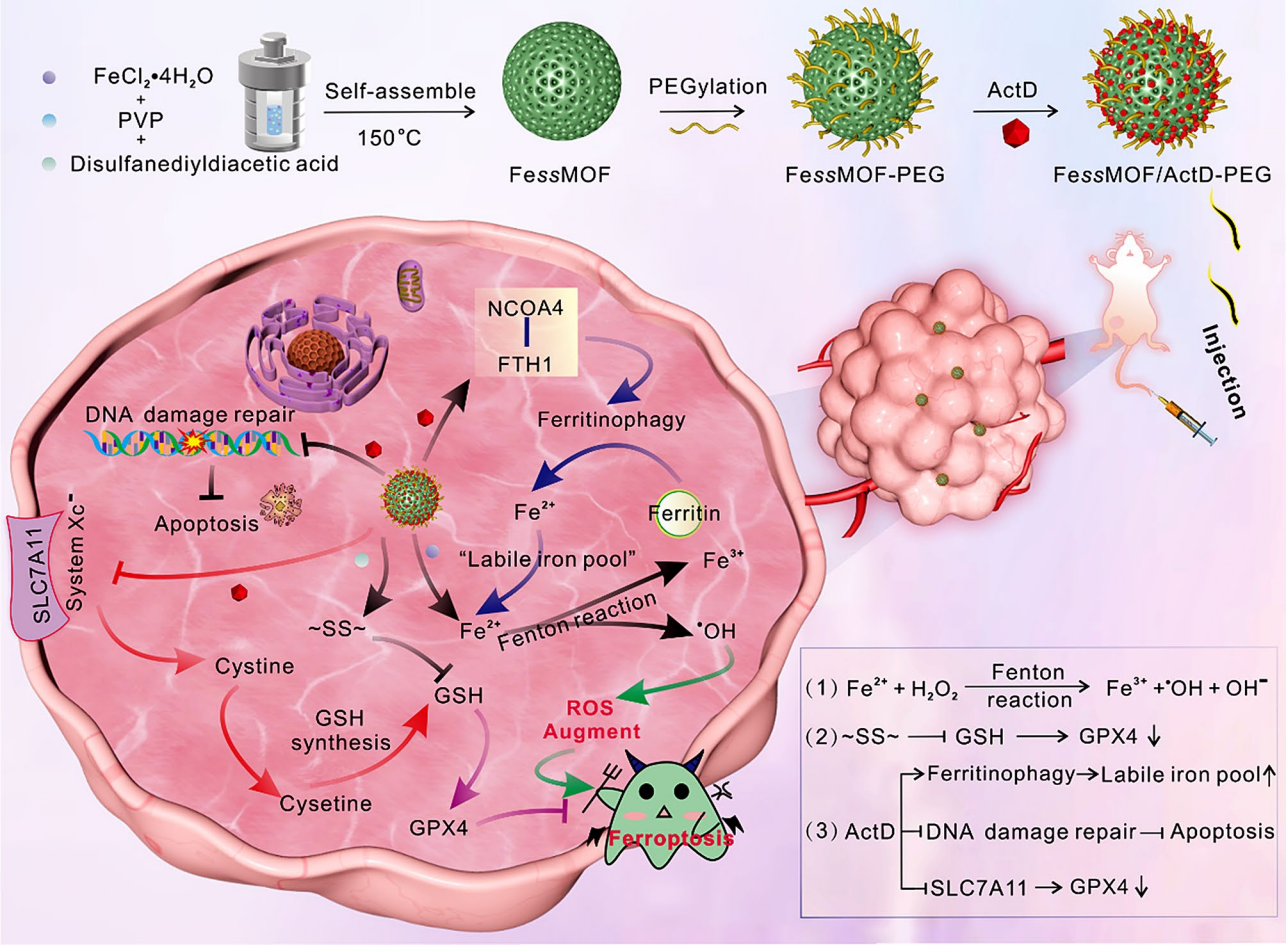
Schematic showing the preparation of Fe*ss*MOF/ActD-PEG and mechanism of ferritinophagy-activated synergistic ferrotherapy. Illustration of the manufacturing procedures of Fe*ss*MOF/ActD-PEG, GSH/pH-stimulated responsive release of the drug, and the action of ActD to damage DNA and enhance ferritinophagy synergistically empowering the ferrotherapy

In this work, we fabricated an iron-based metal-organic framework nanoplatform (Fe*ss*MOF/ActD-PEG) that was assembled from ferrous ions (Fe^2+^ 96.63% and Fe^3+^ 3.37%) and disulfide bonds followed by PEGylation and actinomycin D (ActD) loading for enhanced tumor ferrotherapy (Scheme 1). The Fe*ss*MOF can be targeted to deliver and release ActD responsively in tumor sites due to its small size and GSH/pH-stimulated biodegradability, respectively. Functionally, Fe*ss*MOF is amenable to deplete GSH by the thiol/disulfide exchange reaction to antagonize GPX4 [38]. Besides, the peroxidase (POD)-like activity enables Fe*ss*MOF for tumor-specific catalytic therapy through ^•^OH production and DNA damage in cancer cells. In the meanwhile, ActD can inhibit DNA damage repair to induce cellular apoptosis [39]. Interestingly, we found that the ActD is capable of inhibiting SLC7A11 (Solute Carrier Family 7 Member 11) and inciting ferritinophagy, which further enhances cellular ferroptotic death by restraining GSH synthesis (indirectly inhibits GPX4 expression) and increasing labile iron pool, respectively.

## Results and discussion

### Synthesis and characterization of Fe*ss*MOF/ActD-PEG

Details for the preparation of the Fe*ss*MOF/ActD-PEG nanoplatform are illustrated in Scheme 1. Firstly, Fe*ss*MOF nanoparticles were synthesized by the assembly of disulfide bond-contained dithiodiacetic acid and ferric chloride tetrahydrate in the polyvinylpyrrolidone (PVP, K40) ethanol solution. Then, Fe*ss*MOF nanoparticles were adsorbed with 1, 2-distearoyl-sn-glycero-3-phosphoethanolamine- poly (ethylene glycol) (DSPE-PEG), yielding Fe*ss*MOF-PEG nanoparticles. After loading ActD, the Fe*ss*MOF/ActD-PEG nanoplatform was obtained.

**Fig. 1:**
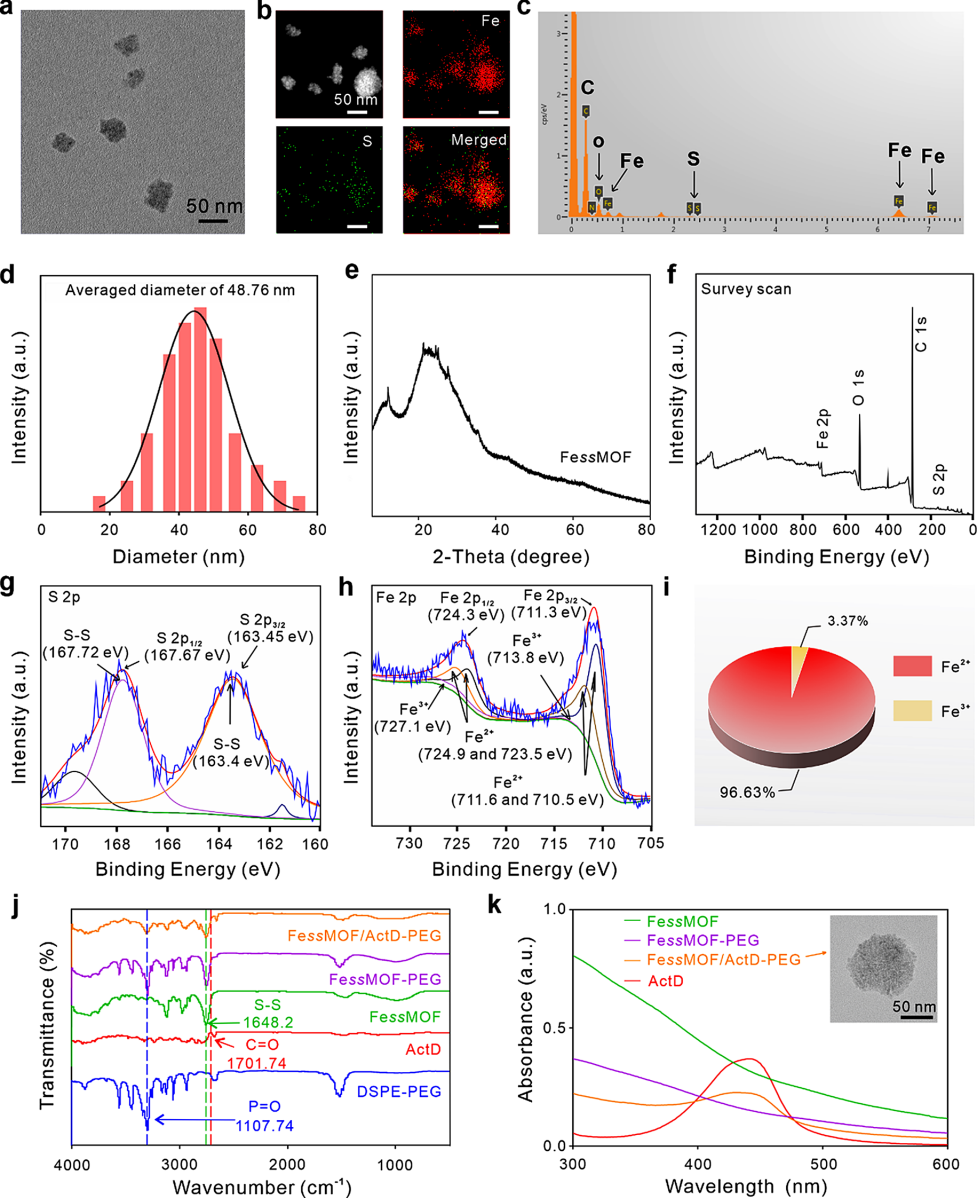
Characterization of Fe*ss*MOF nanoparticles. (**a**) Representative TEM images of Fe*ss*MOF. (**b**) HAADF-STEM and EDX element mapping images of Fe*ss*MOF. (**c**) EDX analysis of FessMOF. (**d**) The average size of Fe*ss*MOF. (**e**) PXRD pattern of Fe*ss*MOF. (**f**) Full range XPS spectra of Fe*ss*MOF, deconvoluted XPS survey of (**g**) S 2p and (**h**) Fe 2p. (**i**) The ratio of the Fe^2+^/Fe^3+^ in Fe 2p. (**j**) FTIR spectra. (**k**) Comparison of UV-vis absorbance of different formulas, inset is the TEM image of Fe*ss*MOF/ActD-PEG nanoplatform

Transmission electron microscopy (TEM) images showed the Fe*ss*MOF nanoparticles hold uniform size and irregular shape (Fig. 1a). The elemental mapping (Fig. 1b) and energy dispersive x-ray spectroscopy (EDX) analysis (Fig. 1c) revealed the elements of S and Fe in Fe*ss*MOF nanoparticles. As shown in Fig. 1d, the size of Fe*ss*MOF nanoparticles is about 50 nm. Powder X-ray diffraction (PXRD) measurement indicated the synthesis of Fe*ss*MOF nanoparticles (Fig. 1e). X-ray photoelectron spectroscopy (XPS) was further carried out to determine the elemental composition and valence. As depicted in Fig. 1f, there are S 2p (163.08 eV), O 1s (532.08 eV), C 1s (285.08 eV), and Fe 2p (711.08 eV) peaks in full XPS scan spectrum, showing that Fe*ss*MOF nanoparticles consisted with elements of S, O, C, and Fe, which was in good consistent with EDX measurement in Fig. 1c. S 2p orbital analysis delineated the disulfide bonds (167.67 and 163.4 eV) in Fe*ss*MOF nanoparticles (Fig. 1g). As can be seen from Fig. 1h, both Fe^3+^ (727.1 and 713.8 eV) and Fe^2+^ (711.6 and 724.9 eV) in the fabricated Fe*ss*MOF nanoparticles. Semiquantitative analysis of Fe 2p spectra manifested the ratio of Fe^2+^ in the iron composition is 96.63% while only 3.37% of Fe^3+^ (Fig. 1i). For the evaluation of the successful PEGylation and drug loading of Fe*ss*MOF nanoparticles, Fourier transform infrared spectroscopy (FTIR) spectrum and UV-vis absorption were investigated. As shown in Fig. 1j, the vibrational peak at 1648.2 cm^− 1^ provides further evidence that the Fe*ss*MOF nanoparticles were assembled from S-S bonds. Moreover, the appearance of P = O (1107.74 cm^− 1^) and C = O (1701.74 cm^− 1^) vibrational peaks in Fe*ss*MOF/ActD-PEG demonstrated the successful PEGylation and ActD loading by Fe*ss*MOF nanoparticles, respectively. The absorption

peak at 440 nm of Fe*ss*MOF/ActD-PEG further informed the ActD was loaded (Fig. 1k). The zeta potential of Fe*ss*MOF nanoparticles with various modifications was illustrated in Fig. S1. And the TEM image (inset of Fig. 1k) indicated the Fe*ss*MOF/ActD-PEG nanoplatform retains an intact morphology compared to the Fe*ss*MOF nanoparticle. Besides, FessMOF exhibited good stability within 5 days in PBS and DMEM medium with 10% FBS solutions (Fig. S2).

**Fig. 2:**
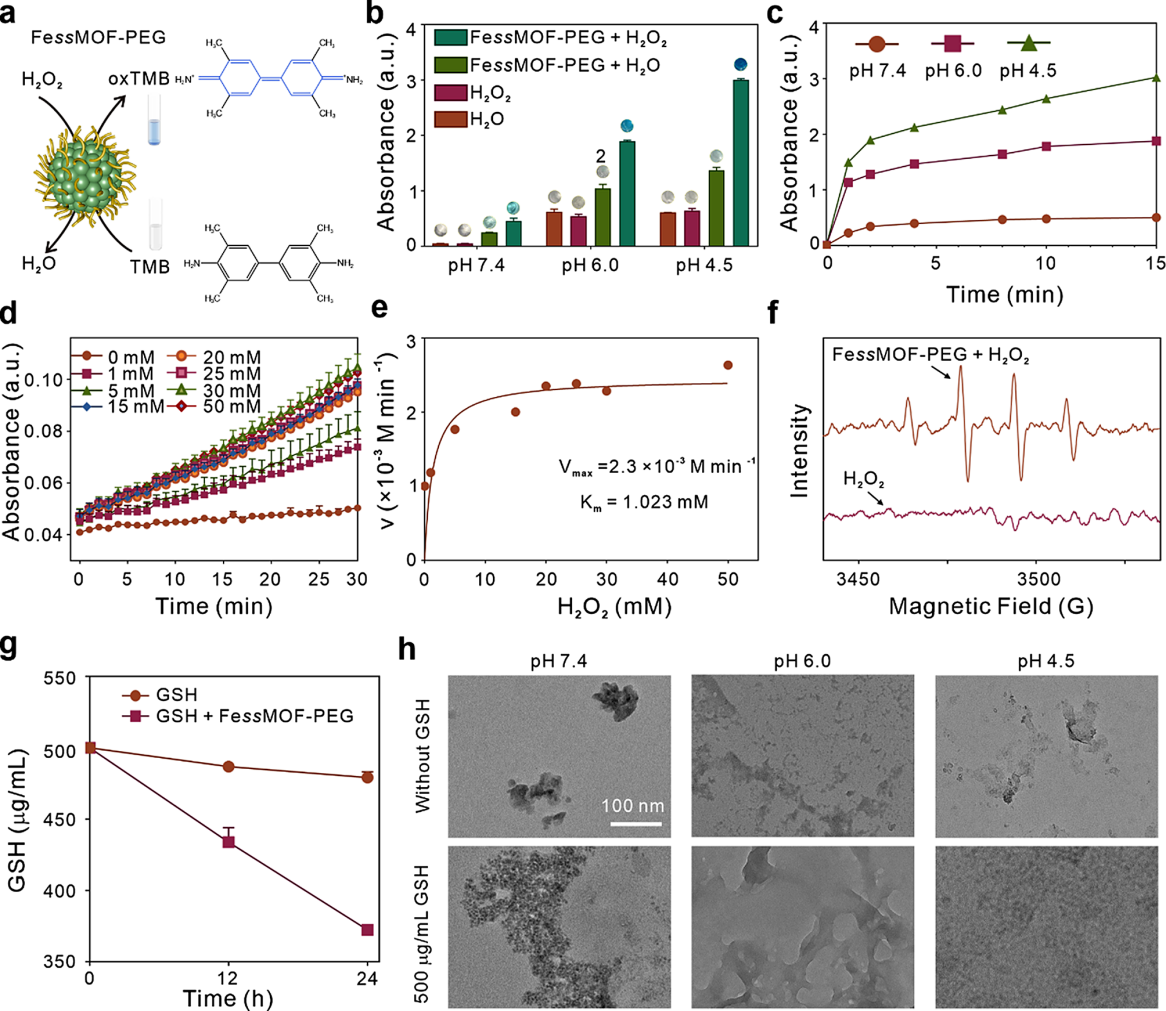
Evaluation of catalytic activities and degradation. (**a**) Schematic diagram of the Fe*ss*MOF-PEG-catalyzed oxidation of TMB (oxTMB) process. (**b**) Absorbance of Fe*ss*MOF-PEG (50 µg/mL) and the catalyzed oxidation of TMB at 15 min under different pH treatments with and without H_2_O_2_ (50 mmol/L), insets present the photos of various mixtures. (**c**) Absorbance change curves of Fe*ss*MOF-PEG (50 µg/mL) and the catalyzed oxidation of TMB at 0–15 min under different pH treatments with and without H_2_O_2_ (50 mmol/L). (**d**) Variation of the time-dependent absorbance values of Fe*ss*MOF-PEG (50 µg/mL) at different concentrations of H_2_O_2_ (0, 1, 5, 15, 20, 25, 30, and 50 mmol/L). (**e**) Michaelis-Menten kinetic analysis for Fe*ss*MOF-PEG with H_2_O_2_ as a substrate. (**f**) ESR spectra of hydroxyl radical trapped by DMPO under different conditions, the concentration of FessMOF-PEG was 100 µg/mL. (**g**) GSH depletion test at 12 h and 24 h. (**h**) TEM images of Fe*ss*MOF-PEG (50 µg/mL) after stirring with GSH (500 µg/mL) under different pH (7.4, 6.0, and 4.5) for 24 h

Next, the POD-mimic activity of Fe*ss*MOF nanoparticles was evaluated by a 3,3’,5,5’-tetramethylbenzidine (TMB) colorimetric assay (Fig. 2a), in which colorless TMB can be oxidized by ^•^OH to bluish oxTMB. Fe*ss*MOF-PEG nanoparticles (50 µg/mL) were incubated with 1% TMB solutions with different pH values (7.4, 6.0, and 4.5). After 15 min, the absorbance of mixtures at 652 nm was measured (Fig. 2b). We found that Fe*ss*MOF-PEG nanoparticles could generate ^•^OH to oxidize TMB and the catalytic activity was enhanced in the presence of H_2_O_2_ (50 mmol/L) and under acidic conditions (Fig. S3). The time-dependent absorbance changes of TMB solutions were shown in Fig. 2c, implying that the weak acidic environment contributes to the enhanced POD activity of the FessMOF-PEG nanoparticles. Furthermore, the steady-state kinetic curves were investigated by varying the H_2_O_2_ concentration (0–50 mmol/L) under neutral conditions (pH 7.4) and measuring the change in absorbance of the reaction system at 652 nm from 0 to 30 min (Fig. 2d). The Michaelis-Menten curves of H_2_O_2_ were obtained by plotting the initial reaction rate versus concentration (Fig. 2e). The Michaelis-Menten constant (K_m_) and the maximum reaction rate (V_max_) were calculated from the double inverse curve (Lineweaver-Burk plot) with K_m_ of 1.023 mmol/L, and V_max_ was 3.83 × 10^− 5^ M/s, much higher than the V_max_ of 8.71 × 10^− 8^ M/s for HRP-catalyzed H_2_O_2_ (Fig. S4, Table S1). In addition, the electron spin resonance (ESR) spectroscopy results using 5,5-dimethyl-1-pyrroline-N-oxide (DMPO) as ^•^OH trapping agent (Fig. 2f) further demonstrated the POD activity of the FessMOF-PEG. Subsequently, the performance of GSH depletion by Fe*ss*MOF-PEG nanoparticles was examined. As can be seen from Fig. 2g, Fe*ss*MOF-PEG consumed 13.4% and 25.5% of GSH after 12 and 24 h, respectively, which confirmed the GSH depletion ability of the disulfide bond-contained Fe*ss*MOF-PEG nanoparticles. Because the exchange reaction between GSH and disulfides may destroy the integrity of disulfide bond-consisted materials, the responsive degradability of Fe*ss*MOF-PEG nanoparticles was studied. We dispersed Fe*ss*MOF-PEG nanoparticles in solutions with various pH values (7.4, 6.0, and 4.5) and were shocked with 500 µg/mL GSH for 24 h. As illustrated in TEM images (Fig. 2h), Fe*ss*MOF-PEG nanoparticles maintained a relatively intact morphology under physiological conditions (pH 7.4) in the absence of GSH. By contrast, the morphology of the nanoparticles disintegrated under acidic conditions (pH 6.0 and 4.5). In addition, GSH promoted the degradation of the Fe*ss*MOF-PEG nanoparticles. All considered, these data suggest that our fabricated nanoparticles not only have POD-like activity and GSH consumption ability but hold pH/GSH-responsive biodegradability, suggesting the potential in tumor-specific catalytic therapy and drug delivery of the as-prepared Fe*ss*MOF-PEG nanoparticles.

### Cell uptake and cytotoxicity

**Fig. 3:**
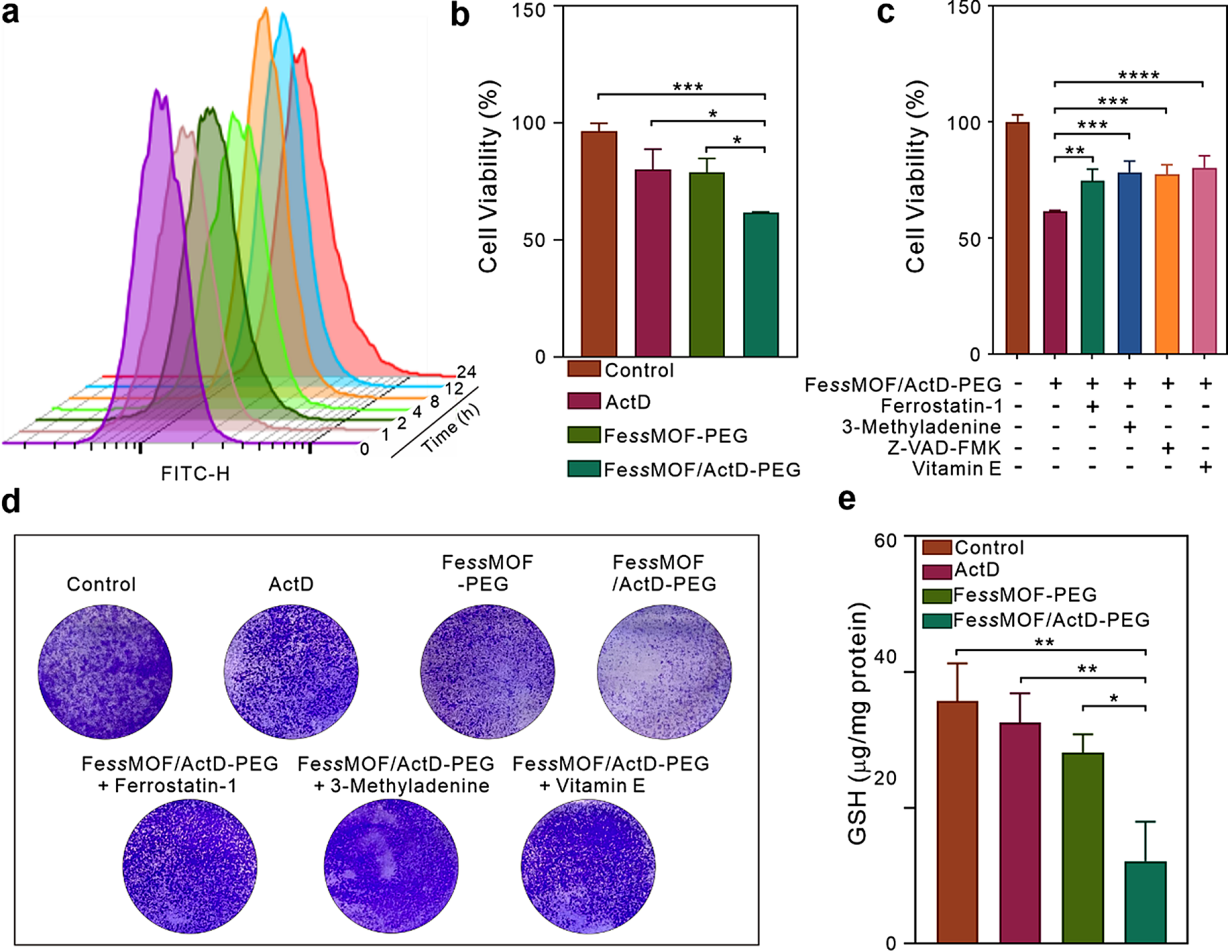
Cellular uptake and in vitro tumor therapy. (**a**) Uptake of 4T1 cells incubated with 50 µg/mL Fe*ss*MOF-PEG@FITC at different time points. (**b**) Cell viability of 4T1 cells after different treatments. (**c**) Cell viability of 4T1 cells cocultured with Fe*ss*MOF-PEG and 10 µM Ferrostatin-1 (ferroptosis inhibitor), 10 µM 3-Methyladenine (autophagy inhibitor), 20 µM Z-VAD-FMK (apoptosis inhibitor), and 20 µM vitamin E (antioxidant) treatments for 24 h. (**d**) Colony formation ability of 4T1 cells under different inhibitors. (**e**) The levels of GSH in the cells upon different treatments. All data were presented as mean ± standard deviation. Statistical differences were calculated using two-tailed Student’s *t* test. Differences were considered significant when the p-value was less than or equal to 0.05. * *p* < 0.05, ** *p* < 0.01, *** *p* < 0.001, and **** *p* < 0.0001

Inspired by the catalytic properties of Fe*ss*MOF-PEG, we closely followed with an experimental investigation of its synergistic anti-tumor properties in vitro in the murine breast tumor 4T1 cell line. We first examined the cellular uptake of Fe*ss*MOF- PEG nanoparticles. Fluorescein isothiocyanate (FITC)-labeled Fe*ss*MOF-PEG nanoparticles were incubated with 4T1 cells for different times and the cellular fluorescence intensity was analyzed by flow cytometry (Fig. 3a). The time-dependent increment of fluorescence signals demonstrated that 4T1 cells could effectively uptake Fe*ss*MOF-PEG. Afterward, the cytotoxicity of Fe*ss*MOF-PEG and ActD were investigated using CCK-8 experiments. According to Fig. S5-S9, a low concentration of ActD has a high cytotoxicity to 4T1 cells. Next, 4T1 cells were incubated with ActD (0.5 ng/mL), Fe*ss*MOF-PEG (50 µg/mL) (Fig. S10), and ActD-loaded Fe*ss*MOF-PEG (ActD, 0.5 ng/mL; Fe*ss*MOF-PEG, 50 µg/mL) for 24 h. As shown in Fig. 3b, Fe*ss*MOF/ActD-PEG nanoplatform exhibited lower cell viability than the ActD or Fe*ss*MOF-PEG, showing that ActD exhibits a synergistic effect on Fe*ss*MOF-PEG nanoparticles for cell killing. Subsequently, the therapeutic mechanism of the Fe*ss*MOF/ActD-PEG nanoplatform was studied by treatment with inhibitors including ferrostatin-1 (ferroptosis), 3-Methyladenine (autophagy), Z-VAD-FMK (apoptosis), and vitamin E (oxidant and ferroptosis). All inhibitors rescued Fe*ss*MOF/ActD-PEG nanoplatforms-induced cell death (Fig. 3c), implying that cell-killing by Fe*ss*MOF/ActD-PEG involved ferroptosis, autophagy, apoptosis, and oxidative stress-mediated ferroptosis, respectively. Practically, clone-formation assay further evidenced the ferroptotic and autophagic cell killing mechanisms of the Fe*ss*MOF/ActD-PEG nanoplatform (Fig. 3d). Subsequently, intracellular GSH levels were tested by a GSH detection kit (Fig. 3e). We found that Fe*ss*MOF-PEG could also consume the cellular GSH. Interestingly, ActD with a low concentration (0.5 ng/mL) has a moderate GSH depletion capacity. And the combination of ActD and Fe*ss*MOF-PEG significantly depleted GSH in 4T1 cells.

### Ferroptosis and ferritinophagy evaluation

**Fig. 4:**
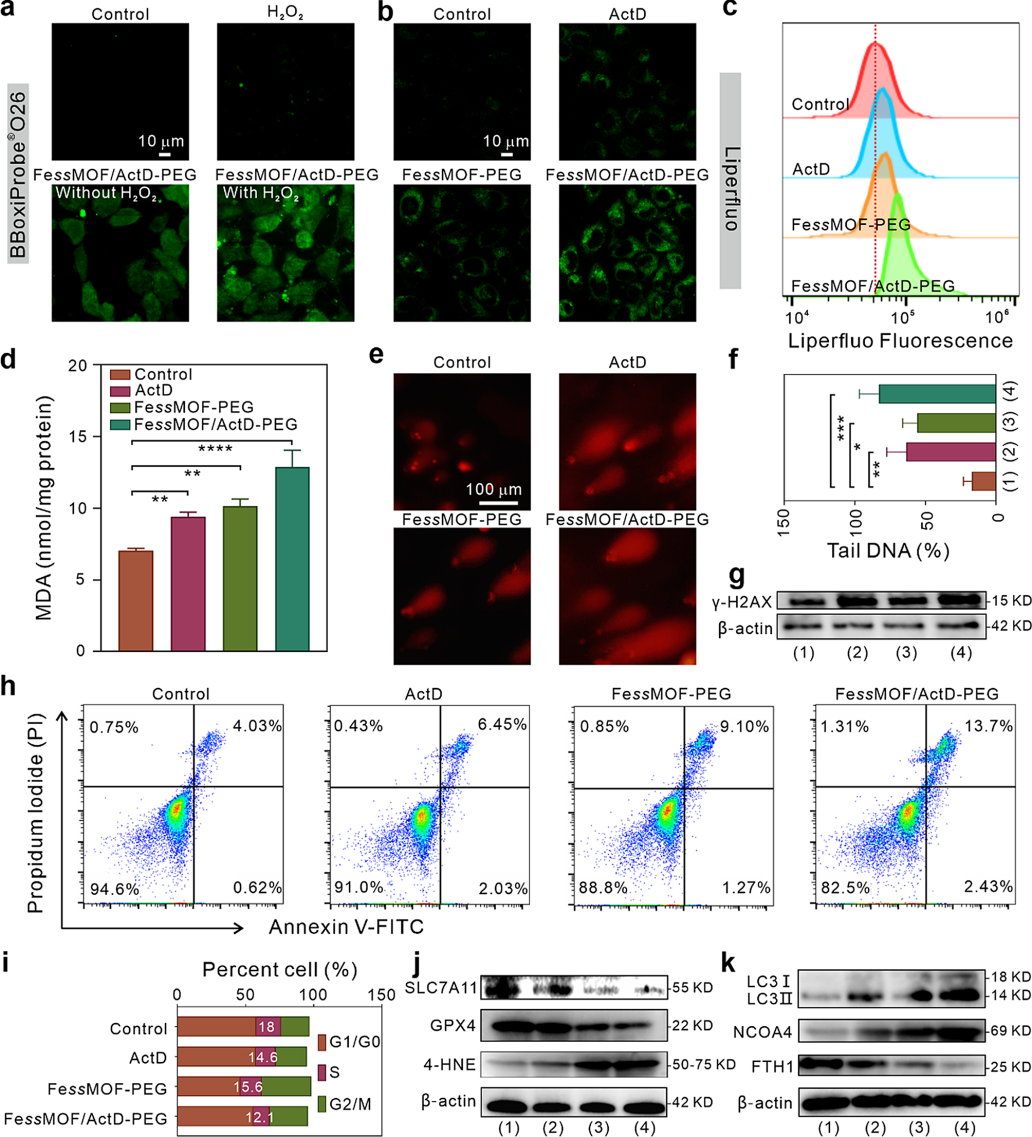
Synergistic ferrotherapy. (**a**) ^•^OH staining by a BBoxiProbe®O26 fluorescent probe in 4T1 cells after different treatments. (**b**) Lipid peroxides imaging by a Liperfluo fluorescent probe. (**c**) Flow cytometry analysis of the LPO fluorescence intensity of 4T1 cells subjected to different treatments. (**d**) Measurement of intracellular MDA levels. (**e**, **f**) Images of cell comet electrophoresis assay (**e**) and percentage of fluorescence intensity (Tail DNA%) in the comet tail (**f**) in 4T1 cells after different treatments. (**g**) Western-blot analysis of the expression of γ-H2AX. (**h**) Flow cytometry analysis of the apoptosis of 4T1 cells after different treatments. (**i**) Quantitative analysis of cell cycle distribution by flow cytometer. (**j**, **k**) Western-blot analysis of the expression of proteins that related to ferroptosis (**j**) and ferritinophagy (**k**). (1), (2), (3), and (4) indicate the groups of control, ActD, Fe*ss*MOF-PEG, and Fe*ss*MOF/ActD-PEG, respectively. All data were presented as mean ± standard deviation. Statistical differences were calculated using two-tailed Student’s *t* test. Differences were considered significant when the p-value was less than or equal to 0.05. * *p* < 0.05, ** *p* < 0.01, *** *p* < 0.001, and **** *p* < 0.0001

It is well-known that nanoparticles with POD-like activity are able to produce highly reactive ^•^OH by Fenton reaction to bring about the accumulation of LPO and DNA damages in tumor cells [40]. Accordingly, we evaluated the ^•^OH generation in 4T1 cells using BBoxiProbe®O26 as the probe. As shown in Fig. 4a, the strong green fluorescence signals in cells that treated with Fe*ss*MOF/ActD-PEG and H_2_O_2_, confirming the efficient ^•^OH generation by the nanoplatform in the presence of the H_2_O_2_. Besides, after the addition of deferoxamine (DFO) (Fig. S11), a classical ferroptosis inhibitor, the ^•^OH generation of FessMOF/ActD-PEG + H_2_O_2_ group significantly reduced, which implies that the iron ions have significant roles in ^•^OH generation. Meanwhile, we found that ActD and Fe*ss*MOF-PEG can trigger moderate LPO generation in 4T1 cells. By comparison, Fe*ss*MOF/ActD-PEG induced significant higher LPO accumulation than ActD and Fe*ss*MOF-PEG (Fig. 4b, c). Next, the lipid peroxidation product malondialdehyde (MDA) was determined (Fig. 4d). It was also found that both ActD and Fe*ss*MOF-PEG nanoparticles elevated the exaggeration of MDA and the Fe*ss*MOF/ActD-PEG induced more MDA accumulation than the ActD and Fe*ss*MOF-PEG. This provides further evidence that both ActD and Fe*ss*MOF-PEG nanoparticles are able to stimulate cell ferroptotic death and it suggests that ActD can promote ferrotherapeutic effects of the Fe*ss*MOF-PEG nanoparticles. Oxidative stress can induce DNA damage and apoptosis while tumor cells tend to repair the damage through various mechanisms thus inhibiting cellular death [41, 42]. Whereas ActD is capable of inhibiting DNA damage repair by intercalating into DNA and interfering with RNA polymerases and DNA topoisomerases, thus inhibiting transcription and inducing various types of DNA damage [39], therefore we used the comet electrophoresis assay to detect DNA damage at the single-cell level. Results in Fig. 4e and f indicated that ActD provoked Fe*ss*MOF-PEG-induced DNA damage in 4T1 cells. In addition, the elevation of the expression of γ-H2AX in Fe*ss*MOF/ActD-PEG- treated cells further confirmed the DNA damage in cancer cells (Fig. 4g). Subsequently, the assay of Annexin V FITC and propidium iodide (PI) co-staining was used to study the cell apoptosis induced by nanoplatforms. As shown in Fig. 4h, the apoptosis ratio (Annexin V FITC ^+ ^and PI^+^) of Fe*ss*MOF/ActD-PEG-treated cells was 13.7% while the ratio of PBS, ActD, and Fe*ss*MOF-PEG was only 4.03%, 6.45%, and 9.10%, respectively. Live/dead staining further confirmed the excellent therapeutic efficacy of the Fe*ss*MOF/ActD-PEG nanoplatform (Fig. S12). In addition, cell cycle arrest analysis demonstrated that Fe*ss*MOF/ActD-PEG lowered the count of cells in the S phase from 18 to 12.1% (Fig. 4i), suggesting Fe*ss*MOF/ActD-PEG is able to inhibit cell DNA replication. These data imply that the as-prepared Fe*ss*MOF/ActD-PEG nanoplatform can induce cell ferroptosis, apoptosis, DNA damage, and cell cycle arrest.

As a classical transcription inhibitor, ActD was found to momentously reduce the half-life of SLC7A11 mRNA, thus may sensitize cells to ferroptosis by repressing expression of SLC7A11 in the Xc^−^ system [43]. It also has been reported that ActD not only can activate cell autophagy and trigger cell apoptosis through inhibiting DNA damage repairment [39, 44]. In the meanwhile, ferroptosis has been identified as a cell death process associated with increased LPO [45]. Therefore, the mechanism of ActD to enhance the ferrotherapeutic effects of the Fe*ss*MOF/ActD-PEG nanoplatform may stem from the SLC7A11 defunctionalization. To validate our hypothesis, we analyzed the expression of the SLC7A11 protein of 4T1 cells treated with ActD, Fe*ss*MOF-PEG, or Fe*ss*MOF/ActD-PEG nanoplatforms. As illustrated in Fig. 4j, ActD exactly reduced the expression of the SLC7A11 protein in cancer cells. And Fe*ss*MOF/ActD-PEG had lower expression of SLC7A11 protein than ActD and Fe*ss*MOF-PEG. Admittedly, the downregulation of the SLC7A11 protein will lead to the reduction of cysteine uptake. While the reduction of cystine uptake further inhibits GSH synthesis and thus suppresses the expression of GPX4 [46, 47], which was evidenced by the western-blot analysis. Consequently, the as-prepared Fe*ss*MOF/ActD-PEG nanoplatform induced pronounced 4-Hydroxynonenal (4-HNE) generation, one of the most important end-products of lipid peroxidation [48]. Furthermore, the elevated expression of LC3II/LC3I and NCOA4 (nuclear receptor coactivator 4) confirmed that ActD can stimulate the ferritinophagy of 4T1 cells (Fig. 4k). Specifically, the Fe*ss*MOF/ActD-PEG nanoplatform induced ferritinophagy of cancer cells and resulted in the degradation of FTH1 (ferritin heavy chain 1), which may increase the labile iron pool and promote cellular ferroptotic death [49]. All considered, we found that ActD inhibits the expression of SLC7A11 and GPX4 and induces cellular ferritinophagy. In the meanwhile, our designed Fe*ss*MOF/ActD-PEG nanoplatform can inhibit DNA damage repair, initiate cell cycle arrest and apoptosis, and trigger robust cellular ferroptosis.

### In vivo biodistribution

**Fig. 5:**
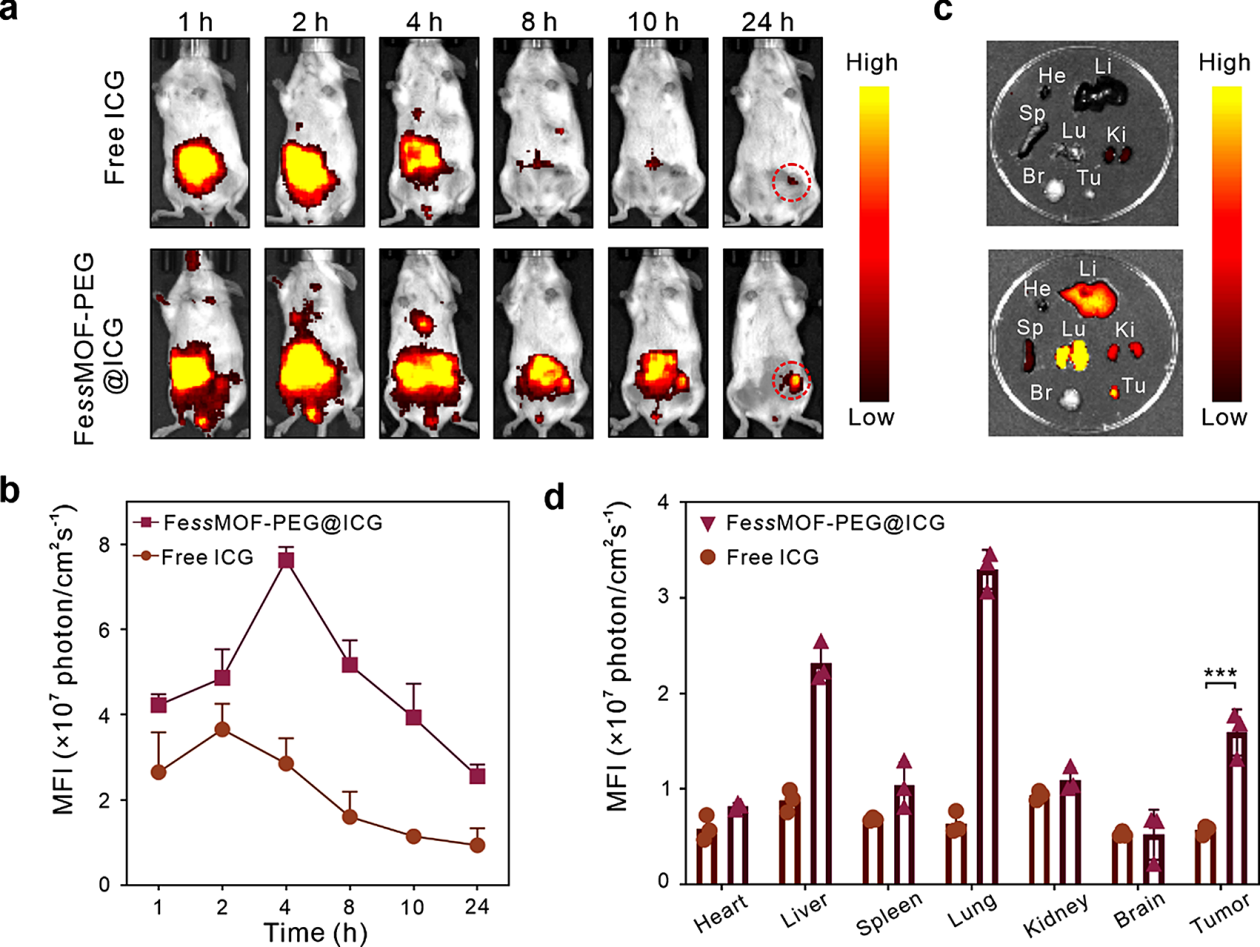
In vivo biodistribution. (**a**) Fluorescence images of 4T1 tumor-bearing mice after intravenous injection of Fe*ss*MOF-PEG@ICG and ICG. (**b**) Comparison of fluorescence intensity at tumor sites upon injection of Fe*ss*MOF-PEG@ICG and ICG. (**c**) Representative fluorescence images of major organs and tumors after intravenous injection of Fe*ss*MOF-PEG@ICG and ICG at 24 h (He, Li, Sp, Lu, Ki, Br, and Tu indicate the heart, liver, spleen, lung, kidney, brain, and tumor, respectively). (**d**) Quantification of fluorescence intensity in tumors and major organs at 24 h. All data were presented as mean ± standard deviation. Statistical differences were calculated using two-tailed Student’s *t* test. Differences were considered significant when the p-value was less than or equal to 0.05. *** *p* < 0.001

Next, the in vivo biodistribution of Fe*ss*MOF-PEG was investigated. 4T1 tumor-bearing mice were injected with ICG-labeled Fe*ss*MOF-PEG (Fe*ss*MOF-PEG@ICG, 40.21% ICG loading rate) (Fig. S13 and Fig. S14) and ICG molecule solutions through the tail vein, and the fluorescence signal was monitored by an imaging system. According to Fig. 5a and b, Fe*ss*MOF-PEG@ICG accumulated at tumor sites. The higher fluorescence signals of Fe*ss*MOF-PEG@ICG than free ICG demonstrated that the Fe*ss*MOF-PEG nanoparticles can improve the tumor-targeting efficiency of therapeutic molecules. After 24 h injection, mice were executed and the tumor and major organs (heart, liver, spleen, lung, kidney, and brain) were imaged. As shown in Fig. 5c and d, the fluorescence intensity of Fe*ss*MOF-PEG@ICG was higher at the tumor site compared to free ICG, suggesting the preferable tumor-targeting property of Fe*ss*MOF-PEG@ICG.

### In vivo antitumor therapy efficacy

**Fig. 6:**
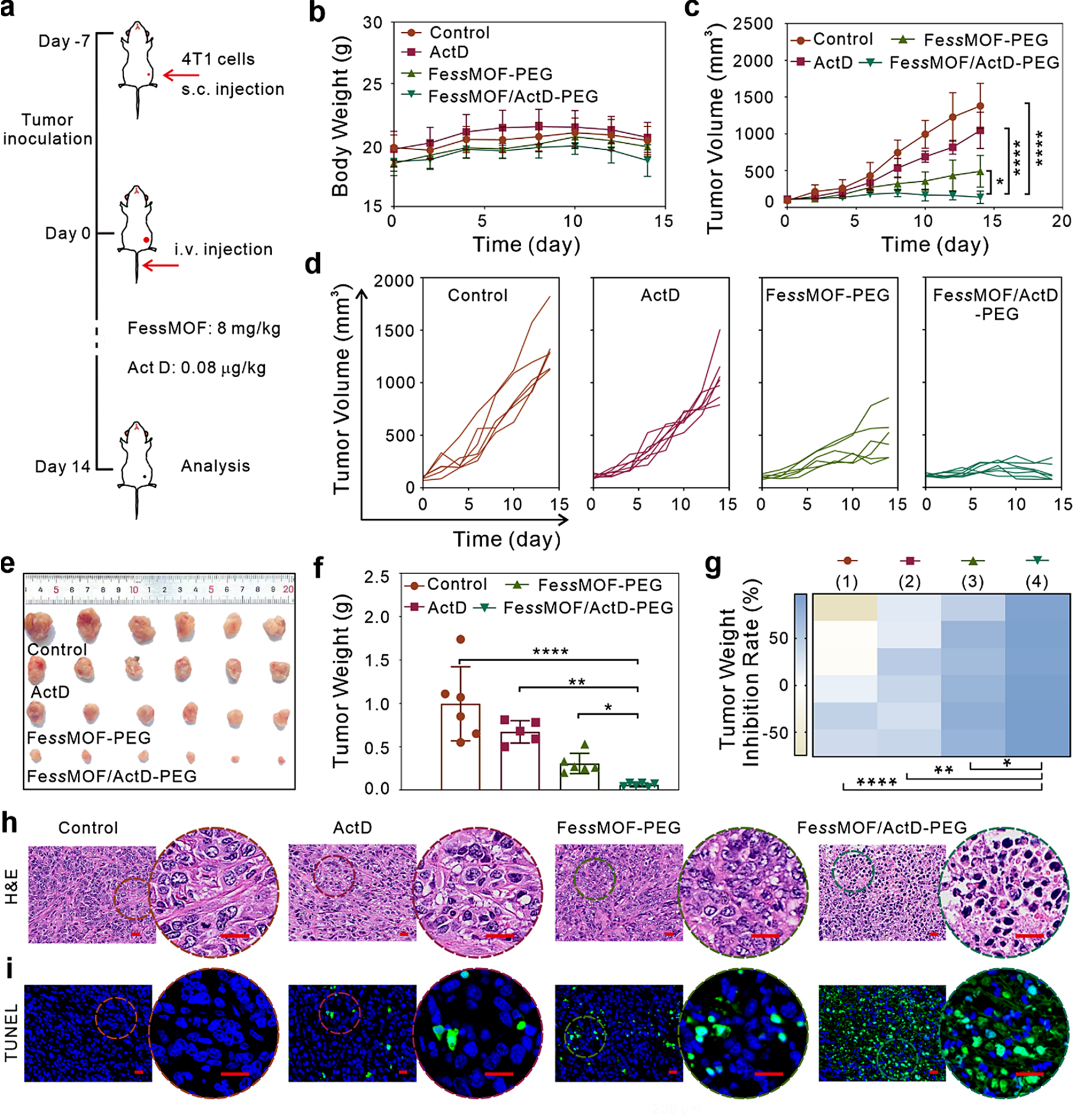
In vivo tumor therapy. (**a**) Scheme illustrating the establishment and treatment of the 4T1 tumor-bearing Balb/c mice. (**b**, **c**) Body weight changes (**b**) and tumor volume (**c**) curves of 4T1 tumor-bearing Balb/c mice after different treatments. (**d**) The records of tumor volume of mice with indicating treatments (*n* = 6). (**e**, **f**) Photographs (**e**) and weights (**f**) of the tumors from different groups after 14 days treatments. (**g**) Percent inhibition rate of tumor weight. (1), (2), (3), and (4) indicate the groups of control, ActD, Fe*ss*MOF-PEG, and Fe*ss*MOF/ActD-PEG, respectively. (**h**, **i**) H&E (**h**) and TUNEL (**i**) staining of tumor slices after treatments. Scale bars are 20 μm. All data were presented as mean ± standard deviation. Statistical differences were calculated using two-tailed Student’s *t* test. Differences were considered significant when the p-value was less than or equal to 0.05. * *p* < 0.05, ** *p* < 0.01, and **** *p* < 0.0001

Given the outstanding in vitro synergistic therapeutic efficacy and tumor-targeting, the in vivo antitumor effects of the Fe*ss*MOF/ActD-PEG nanoplatform were next examined (Fig. 6a). 4T1 tumor-bearing mice were randomly divided into four groups and intravenously injected with PBS (control), ActD, Fe*ss*MOF-PEG, and Fe*ss*MOF/ActD-PEG, respectively. The body weight and tumor volume of the mice were recorded every other day. As can be seen from Fig. 6b and Fig. S15, the body weight of mice had no obvious changes, reflecting the favorable biocompatibility of nanoparticles. Tumors in group control grew rapidly while ActD had a slight effect the growth of the tumor (Fig. 6c, d). By contrast, Fe*ss*MOF-PEG delayed the growth of tumors. Specifically, the group Fe*ss*MOF/ActD-PEG showed a significant tumor inhibition effect and its tumor volume inhibition rate was 91.89% (Fig. S16a). Mice were executed after treatments for 14 days, and tumors from different groups were imaged and weighted (Fig. 6e, f). It also found that the Fe*ss*MOF/ActD-PEG nanoplatform had a higher inhibition efficiency than the other groups (Fig. 6g). H&E (hematoxylin and eosin) histologic staining (Fig. 6h) and TUNEL (terminal deoxynucleotidyl transferase-mediated dUTP Nick-End labeling) immunofluorescent staining (Fig. 6i and Fig. S16b) of tumor slices illustrated that more cell death and damage was found in group Fe*ss*MOF/ActD-PEG after treatments. All these results demonstrated that our designed Fe*ss*MOF/ActD-PEG nanoplatform exhibited pronounced therapeutic effects on tumor therapy.

### In vivo biosafety evaluation

**Fig. 7:**
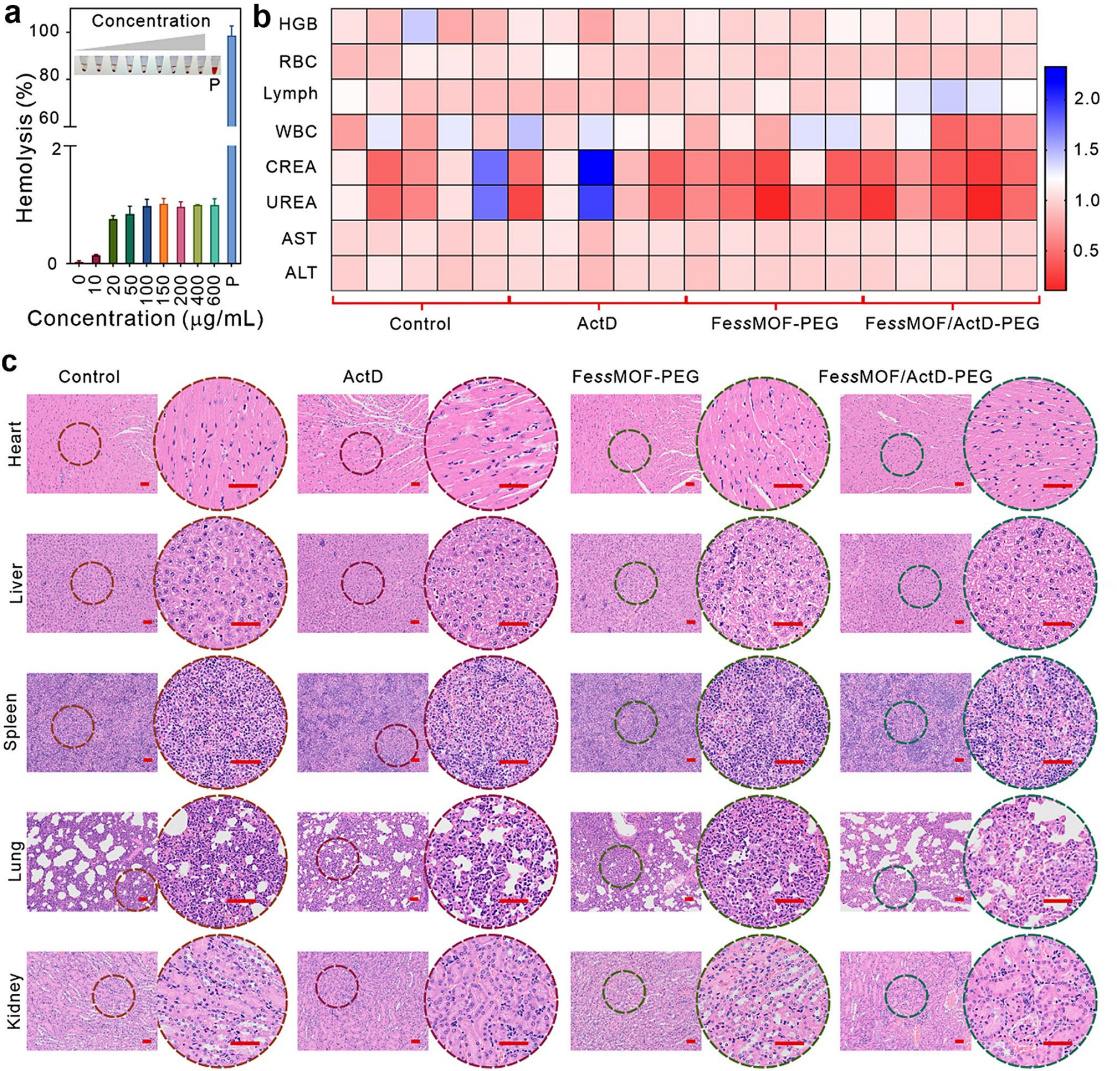
Evaluation of biocompatibility. (**a**) Hemolysis rate of Fe*ss*MOF-PEG with different concentrations. TritonX-100 (0.5%) as the positive control (P), inset is the photo of red blood cell solution after incubating with Fe*ss*MOF-PEG nanoparticles. (**b**) Hematological indexes and biochemical data of 4T1 tumor-bearing Balb/c mice after treatments for 14 days. HGB, RBC, Lymph, WBC, CREA, UREA, AST, and ALT indicate hemoglobin, red blood cell, lymphocyte, white blood cell, creatinine, urea, aspartate transaminase, and alanine transaminase, respectively. (**c**) H&E staining of major organs from treated mice. Scale bars are 50 μm

The biosafety of nanomaterials is one of the major concerns in biomedical applications. To investigate the biosafety of the Fe*ss*MOF-PEG nanoparticles, we first performed the hemolysis assay (Fig. 7a). We found that the hemolysis rate was less than 1% even though the concentration of Fe*ss*MOF-PEG nanoparticles was up to 600 µg/mL, which demonstrates the excellent biocompatibility of the synthesized nanoparticles. After 14 days of treatments, blood and major organs from mice were collected. According to the blood and biochemical analysis, Fe*ss*MOF/ActD-PEG has no noticeable fluctuation in the correlation index compared to other groups (Fig. 7b). Additionally, no significant cell necrosis or lesions were found in the major organs (heart, liver, spleen, lung, and kidney) of mice after treatments (Fig. 7c). These data suggest that the as-prepared Fe*ss*MOF/ActD-PEG nanoplatform exhibits excellent in vivo biocompatibility.

## Conclusion

In summary, we have demonstrated the construction of a ferrous-based MOF nanoplatform that is assembled from ferrous ions and disulfide bonds for tumor microenvironment-responsive ferrotherapy. The Fe*ss*MOF nanoparticles hold POD and GSH oxidase dule enzyme-like activities, which could alleviate cellular oxidative stress and suppress the GPX4. Combined with the ActD, the Fe*ss*MOF/ActD-PEG nanoplatform induced more LPO generation, DNA damage, and cell cycle arrest than the Fe*ss*MOF nanoparticle or free ActD. By taking full functions of ActD and Fe*ss*MOF, the as-prepared nanoplatform results in a pronounced ferroptosis-mediated synergistic therapeutic efficacy both in vitro and in vivo. Additionally, Fe*ss*MOF/ActD-PEG exhibits excellent biocompatibility and low side effects. In a nutshell, our work reported a smart ferroptosis nanoinducer for tumor-specific therapy, which may offer new insight into developing novel nanoplatforms for tumor ferrotherapy in the future.

## Materials and methods

### Materials

Polyvinylpyrrolidone (PVP, K40) was obtained from Sigma Aldrich. N, N-dimethylformamide (DMF), dithiodiglycolic acid, iron (II) chloride tetrahydrate, triethanolamine (TEA), ferrostatin-1, indocyanine green (ICG), and D-α-tocopherol were purchased from Shanghai Aladdin Biochemical Technology Co., Ltd. Actinomycin D (ActD) was obtained from MedChemExpress. DSPE-mPEG was acquired from Shanghai Yaye Gaoyuan Enterprise Management Co., Ltd. CCK8 kit and cell culture medium were received from YENSEN Technology Co., Ltd. 3-Methyladenine was bought from Shanghai Macklin Biochemical Co., Ltd. Necrostatin-1, Calcein-AM, propidium iodide (PI), BeyoClickTMEdU-555 Cell proliferation test kit, and Z-VAD-FMK were purchased from Beyotime Biochemical Co., Ltd. GSH assay kit and 1% TMB solution were purchased from Solarbio Science & Technology Co., Ltd. BBoxiProbe® HPF Hydroxyl Radical Probe was gained from Shanghai BestBio Biochemical Co., Ltd. Beta actin (β-actin) monoclonal antibody, LC3 polyclonal antibody, GPX4 monoclonal antibody, HRP-conjugated Affinipure Goat Anti-Rabbit IgG (H + L), HRP-conjugated Affinipure Goat Anti-Mice IgG, and SLC7A11/xCT antibody were got from Proteintech. 4-HNE antibody was obtained from Origo Biopharma Co., Ltd. FTH1 antibody and NCOA4 antibody were acquired from Shanghai absin Biochemical Co., Ltd. Liperfluo and Annexin V-FITC/PI apoptosis detection kit were gained from Dojindo Molecular Technologies, Inc. Fetal bovine serum (FBS) was got from PAN. All reagents were used as received.

### Synthesis of FessMOF/ActD-PEG

Fe*ss*MOF nanoparticles were synthesized at first according to a reported work with some modifications [27]. In detail, 300 mg PVP (K40) was thoroughly ultrasonically dispersed in 3 mL DMF. Then, dithioglycolic acid (52 µL, 100 mg/mL), FeCl_2_•4H_2_O (7 mg), triethanolamine (600 µL), and 10 mL of a mixed solution of DMF and anhydrous ethanol (the volumetric ratio of DMF and anhydrous ethanol is 5: 3) were added into the above PVP solution under stirring. After thoroughly mixed, the mixture was transferred to a Teflon-lined stainless-steel autoclave and kept at 150 °C for 12 h. The sediments were centrifuged (11,000 rpm, 15 min) and washed several times with water. Next, Fe*ss*MOF nanoparticles were absorbed with DSPE-mPEG. Fe*ss*MOF nanoparticles (1 mg) were mixed with DSPE-mPEG (1 mg) in 1 mL distilled water for 6 h at 4 °C. Afterward, Fe*ss*MOF-PEG nanoparticles were obtained by centrifugation. Finally, Fe*ss*MOF-PEG nanoparticles (1 mg) were added into the 1 mL ActD solution (0.5 ng/mL) overnight at 4 °C, and the final precipitates were washed with distilled water, obtaining the Fe*ss*MOF/ActD-PEG nanoplatform. The loading efficiency of ActD in the Fe*ss*MOF-PEG was calculated as (input ActD quantity - supernatant ActD quantity) / input ActD quantity × 100%. The final load rate of ActD is about 100%.

### Characterization

Transmission electron microscopy (TEM) images were obtained by HT7700 Exalens transmission electron microscope. Zeta potential and dynamic light scattering (DLS) were measured on the Malvern Zetasizer Nano ZS (Malvern Instruments, Ltd., Worcestershire, UK). X-ray photoelectron spectroscopy (XPS) spectra were acquired by the Thermo SCIENTIFIC Nexsa a K-Alpha 1063 instrument (Thermo Fisher Scientific, USA). Fourier transform infrared spectroscopy (FTIR) spectra were tested on an FTIR spectrometer (ThermoNicolet IS50). UV-vis spectra were measured by UV-8000 S spectrophotometer. X-ray Diffraction (XRD) analysis of samples was conducted by a Rigaku Miniflex600 X-ray diffractometer.

### Hemolysis experiment

Fresh blood was obtained from Balb/c mice through eyeballs. Red blood cells were centrifuged and washed 3 times with PBS solution. Then the red blood cells were diluted with 5 mL PBS. Preparation of positive control solution: 50 µL diluted mice blood + 50 µL 0.5% Triton-100; preparation of negative control solution: 50 µL diluted mice blood + 50 µL PBS solution; preparation of test group solutions: 50 µL diluted mice blood + different volumes of 1 mg/mL Fe*ss*MOF-PEG. The final concentrations of Fe*ss*MOF-PEG were 10, 20, 50, 100, 150, 200, 400, and 600 µg/mL. After incubation for 60 min at 37 °C on a shaker, the mixture was centrifugated at 3000 rpm for 3 min. Then the supernatant was placed in a 96-well plate, and the absorbance values were measured by a microplate reader (adjusted to 576 nm). The hemolysis rate (%) was measured by the hemolysis rate formula: hemolysis rate (%) = [(absorbance value of the test group - absorbance value of the negative control group) / (absorbance value of the positive control group - absorbance value of the negative control group)] × 100%.

### ESR detection

For ESR detections, the PBS solution (pH 6.0) containing Fe*ss*MOF-PEG (100 µg/mL) and 50 mM H_2_O_2_ were incubated for 15 min at room temperature. After that, the DMPO (100 mM) as the spin-trapping agent was added to the sbove mixture.

### The POD-like activity of FessMOF-PEG

The POD-like activity of Fe*ss*MOF-PEG was evaluated by the TMB colorimetric assay. In detail, 50 µg/mL Fe*ss*MOF-PEG was dispersed in buffer solutions with different pH values (7.4, 6.0, and 4.5). Then, H_2_O_2_ solution (50 mmol/L) and 1% TMB solution were added. After 15 min, the absorbance values at 652 nm of various groups were immediately measured using a microplate reader (Thermo Fisher Scientific).

### GSH consumption evaluation

Fe*ss*MOF-PEG nanoparticles (50 µg/mL) were incubated with GSH (500 µg/mL) at 37 °C for 12 and 24 h. The GSH solution (500 µg/mL) without Fe*ss*MOF-PEG nanoparticles as the control. GSH concentration was measured by a GSH detection kit.

### Degradation of FessMOF-PEG

Fifty µg/mL Fe*ss*MOF-PEG nanoparticles were dispersed in buffer solutions with different pH values (7.4, 6.0, and 4.5) with or without 500 µg/mL GSH solution. After slowly shaking for 24 h at 37 °C, the morphology of Fe*ss*MOF-PEG nanoparticles was observed using transmission electron microscopy.

### Cellular uptake

4T1 cells were seeded in 6-well plates at a density of 10^5^ cells for 24 h and then co-incubated with 50 µg/mL Fe*ss*MOF-PEG@FITC (FITC, 10 µg/mL) for different times (0, 1, 2, 4, 8, 12, and 24 h). Afterward, the fluorescence intensity of FITC in cells was assessed by flow cytometer (NovoCyte Flow Cytometer).

### Cell viability

4T1 cells were seeded in 96-well plates with 10^4^ cells per well for 24 h, and then treated with various treatment solutions, (a) PBS; (b) ActD (0.5 ng/mL); (c) Fe*ss*MOF-PEG (50 µg/mL); (d) Fe*ss*MOF/ActD-PEG (50 µg/mL, containing 50 µg/mL Fe*ss*MOF-PEG and 0.5 ng/mL ActD) for 24 h. The CCK8 assay kit was used to evaluate cell viability. Each well of the 96-well plate received 10 µL of CCK-8 solution. After 1 h of incubation, the cell absorbance values at 450 nm were measured using a microplate reader.

### Colony-forming assay

4T1 cells were seeded in 6-well plates at a density of 1000 cells per well and grew for 24 h. The cells were incubated with various treatment solutions for 24 h. The medium was changed every three days under the same treatment conditions. The co-incubation was terminated after 14 days when colonies were visible to the naked eye. Thereafter, the cells were washed three times with PBS, then fixed with 4% paraformaldehyde for 30 min, rinsed twice with running water, and stained for 30 min with crystal violet solution. Finally, the crystal violet solution was carefully washed away with running water, and the 6-well plates were inverted and naturally dried. Photographs were taken and videotapes were made.

### ^•^OH detection

4T1 cells were seeded in confocal dishes at a density of 10^4^ cells per well and grew for 24 h. After 4T1 cells were incubated with various treatment solutions for 24 h. Diluted BBoxiProbe®O26 probe solution was added into confocal dishes. Then, the cells were washed twice with PBS and imaged by a confocal laser scanning microscopy (LSM780, Zeiss).

### Liperfluo

For cellular LPO evaluation, 4T1 cells were incubated with various treatment solutions for 24 h. Afterward, the cell culture medium was replaced with 10 µmol/L liperfluo probe solution and cultured for 30 min at 37 °C. At last, the cells were imaged under a confocal laser scanning microscopy. Simultaneously, the fluorescence intensity was assessed by a flow cytometer.

### Live/dead assay

After various treatments, 4T1 cells were co-incubated with Calcein-AM (2 µmol/L) and PI (5 µmol/L) staining reagents for 2 h and subsequently imaged by a confocal laser scanning microscopy.

### GSH and MDA detection

4T1 cells were cultured in 6-well plates with a density of 10^6^ cells per well and co-cultivated with (a) PBS; (b) ActD (0.5 ng/mL); (c) Fe*ss*MOF-PEG (50 µg/mL); (d) Fe*ss*MOF/ActD-PEG (50 µg/mL, containing Fe*ss*MOF-PEG 50 µg/mL and ActD 0.5 ng/mL). After 24 h, the GSH and MDA content of each group were measured by the GSH/MDA detection kit according to the manufacturer’s instructions.

### Comet electrophoresis experiment

The experiment was conducted according to the kit instructions (KGI Bio). In detail, the layer of 1% normal melting point gel was configured on the slides. Then, 10 µL of cells solution (about 10^4^ cells) and 75 µL of 0.7% low melting point gel were mixed well and spread on the pretreated slides at 4°C for 10 min to allow the agarose to solidify. Afterward, the glued slides were lysed for 90 min in a freshly prepared lysis buffer. Subsequently, the slides were rinsed in PBS solution before being placed in an electrophoresis buffer, denatured for 30 min, and electrophoresed for 20 min (25 V, 300 mA). And then, neutralization was performed three times for 5 min each with 400 mmol/L tris buffer (pH 7.5). At last, each slide was stained for 20 min with 20 µL of PI staining solution and observed under a Zeiss fluorescence microscope.

### Apoptosis assay

4T1 cells were pre-seeded in 6-well plates at a density of 10^5^ cells per well. The cells were incubated with various treatment solutions for 24 h. Afterward, the adherent cells in 6-well plates were digested with trypsin, washed twice with pre-cooled PBS solution, and finally collected 5 × 10^5^ cells in tubes. Each tube was incubated with 5 µL Annexin V -FITC and 10 µL PI staining solutions for 10 min at 4 °C. Detection on flow cytometer: upper right quadrant (Annexin V^+^/PI^+^, late apoptotic cells); lower right quadrant (Annexin V^+^/PI^-^, early apoptotic cells), upper left quadrant (Annexin V^-^/PI^+^, dead cells), and lower left quadrant (Annexin V^-^/PI^-^, live cells).

### Cell cycle assay

The cultivated 4T1 cells were tagged with EdU. Then, cells were fixed and permeabilized with a BeyoClickTMEdU-555 kit. The results were analyzed by a flow cytometer.

### Western blot

4T1 cells were pre-seeded in a 6-well culture plate. Cells were washed twice with PBS solution and then collected using cell lysate, PMSF, and protease inhibitor. The contents of protein were measured using the BCA protein concentration assay kit. According to the required sample volume, 5 × loading buffer was added into the protein solution at a ratio of 4: 1. Then the final protein solution was denatured at 100 ℃ for 10 min in a boiling water bath. The separation gel and concentrated gel were prepared, and the samples were loaded at 30 µg of protein volume, electrophoretically separated and transferred into the polyvinylidene fluoride (PVDF) membrane, and then closed by 5% skim milk solution at room temperature for 2 h. These samples were incubated with the corresponding primary antibody such as β-actin (1: 20,000, Proteintech), SLC7A11 (1: 1000, Origo), GPX4 (1: 1000, Proteintech), 4- HNE (1: 1000, Origo), LC3 (1: 1000, Proteintech), NCOA4 (1: 1000, Proteintech), FTH1 (1: 1000, Proteintech), and γ-H2AX (1: 1000, Proteintech) overnight at 4 °C. After that, these samples were cultivated with the secondary antibody (1: 10,000, Proteintech) for 2 h. At last, the membranes were visualized and processed with image J for grayscale values.

### In vivo biodistribution

Female Balb/c mice (4 weeks) were purchased from Shanghai Slack Laboratory Animal Co., Ltd., and the experiments were implemented in accordance with protocols approved by the Animal Experimental Ethics Committee of Fujian Normal University.

4T1 tumor models were obtained by orthotopically injecting 5 × 10^6^ 4T1 cells in the breast fat pads of mice. When the tumor volume approached about 80 mm^3^, the eight tumor-bearing mice were randomly divided into two groups. Then, mice were intravenously injected with free ICG or Fe*ss*MOF-PEG@ICG (containing 100 µg ICG per mouse). Subsequently, fluorescent signals of mice were acquired by an imaging spectrum system (PerkinElmer) at different time points (1, 2, 4, 8, 10, and 24 h). After injection for 24 h, mice were euthanized and tumors and major organs (heart, liver, spleen, lung, kidney, and brain) were collected. The ex vivo fluorescence images and intensities of tumors and major organs were recorded by the imaging system.

### In vivo tumor therapy

4T1 cells (5 × 10^6^) were orthotopically injected into the breast fat pads of female Balb/c mice. When the tumor volume reached approximately 100 mm^3^, these mice were injected intravenously with (a) PBS; (b) ActD (0.8 µg/kg); (c) Fe*ss*MOF-PEG (8 mg/kg); (d) Fe*ss*MOF/ActD-PEG (8 mg/kg, containing 8 mg/kg Fe*ss*MOF-PEG and 0.8 µg/kg ActD). Afterward, the tumor volumes and body weights were recorded every other day. The tumor volumes (mm^3^) were measured according to V = *ab*^2^/2, in which *a* and *b* represent the length and width of the tumor, respectively. After 14 days, these mice were executed and their tumors and organs (heart, liver, spleen, lung, and kidney) were collected. Tumor slices were conducted with H&E staining and TUNEL staining. Slices of organs were stained with H&E for damage evaluation. In addition, blood solutions of treated mice were harvested for blood routine analysis including hemoglobin (HGB), red blood cells (RBC), lymphocytes (Lymph), and red blood cells (WBC). In the meanwhile, glutamic aminotransferase (ALT), aspartate aminotransferase (AST), creatinine (CREA), and UREA in plasmas were measured to evaluate the hepatic and renal functions.

### Quantification and statistical analysis

Quantitative data were presented as mean ± standard error of the mean (SEM). Statistics were analyzed by one-way or two-way analysis of variance (ANOVA), followed by two-sided Student’s t-test using GraphPad Prism 5.0. Differences were considered significant when the p-value was less than or equal to 0.05. * *p* < 0.05, ** *p* < 0.01, *** *p* < 0.001, and **** *p* < 0.0001.

### Electronic supplementary material

Below is the link to the electronic supplementary material.

Supplementary Material 1

## Data Availability

The datasets are available from the corresponding author on reasonable request.
