# Supplementary material for: Iron (II)-based metal-organic framework nanozyme for boosting tumor ferroptosis through inhibiting DNA damage repair and system Xc -
Source: J Nanobiotechnology. 2024 May 8;22:228. doi: 10.1186/s12951-024-02508-2 (PMC11077818; doi:10.1186/s12951-024-02508-2)
Supplement: Supplementary file 1 — Supplementary Material 1 [file 12951_2024_2508_MOESM1_ESM.docx]

Supporting Information

**Iron (II)-based metal-organic framework nanozyme for boosting tumor ferroptosis through inhibiting DNA damage repair and system Xc^-^**

Panpan Xue, Huilan Zhuang, Tingjie Bai, Xuemei Zeng^2^*, Jinpeng Deng, Sijie Shao, Shuangqian Yan^1^*

^1^The Straits Institute of Flexible Electronics (SIFE, Future Technologies), Fujian Normal University, The Straits Laboratory of Flexible Electronics (SLoFE), Fuzhou 350117, Fujian, China

^2^Key Laboratory of Innate Immune Biology of Fujian Province, Biomedical Research Center of South China, College of Life Sciences, Fujian Normal University, 1 Keji Road, Fuzhou 350117, PR China

* Corresponding authors.

E-mail: [xmzeng@fjnu.edu.cn](mailto:xmzeng@fjnu.edu.cn), [ifeshqyan@fjnu.edu.cn](mailto:QBX20220169@yjs.fjnu.edu.cn)

**KEYWORDS:** Metal organic framework; Disulfide bond; Autophagy; Ferroptosis;


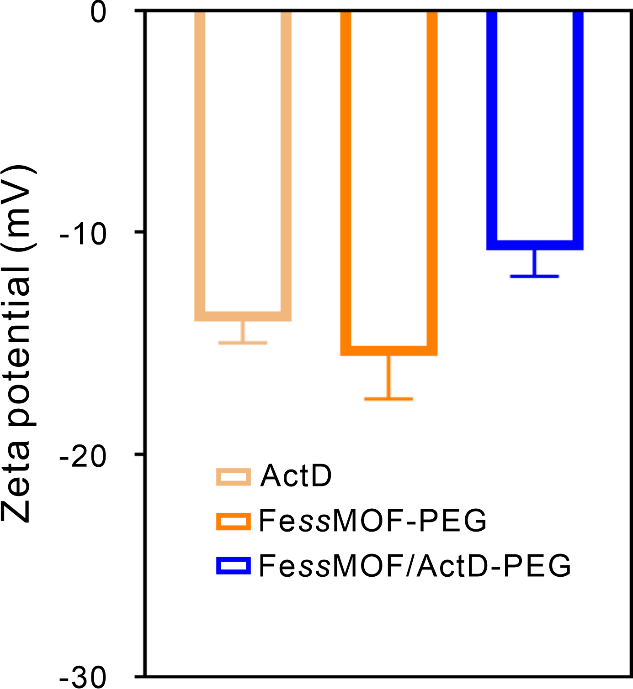


**Fig. S1**. The zeta potential of ActD, FessMOF-PEG, and FessMOF/ActD-PEG.

**
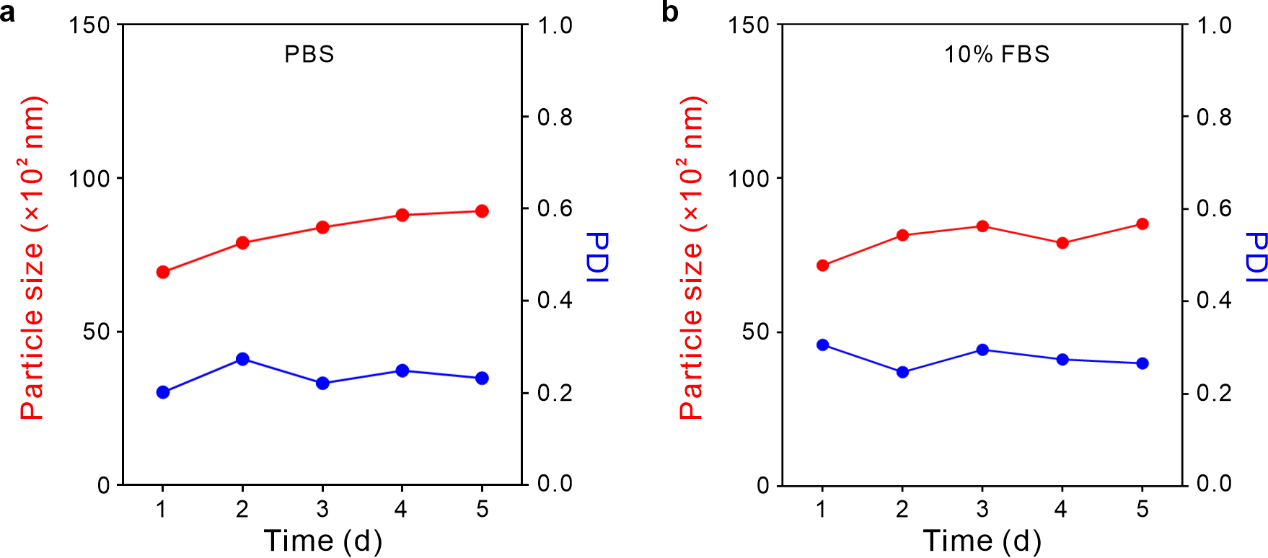
**

**Fig. S2**. The stability of FessMOF in PBS (a) and DMEM medium with 10% FBS (b) within 5 days.


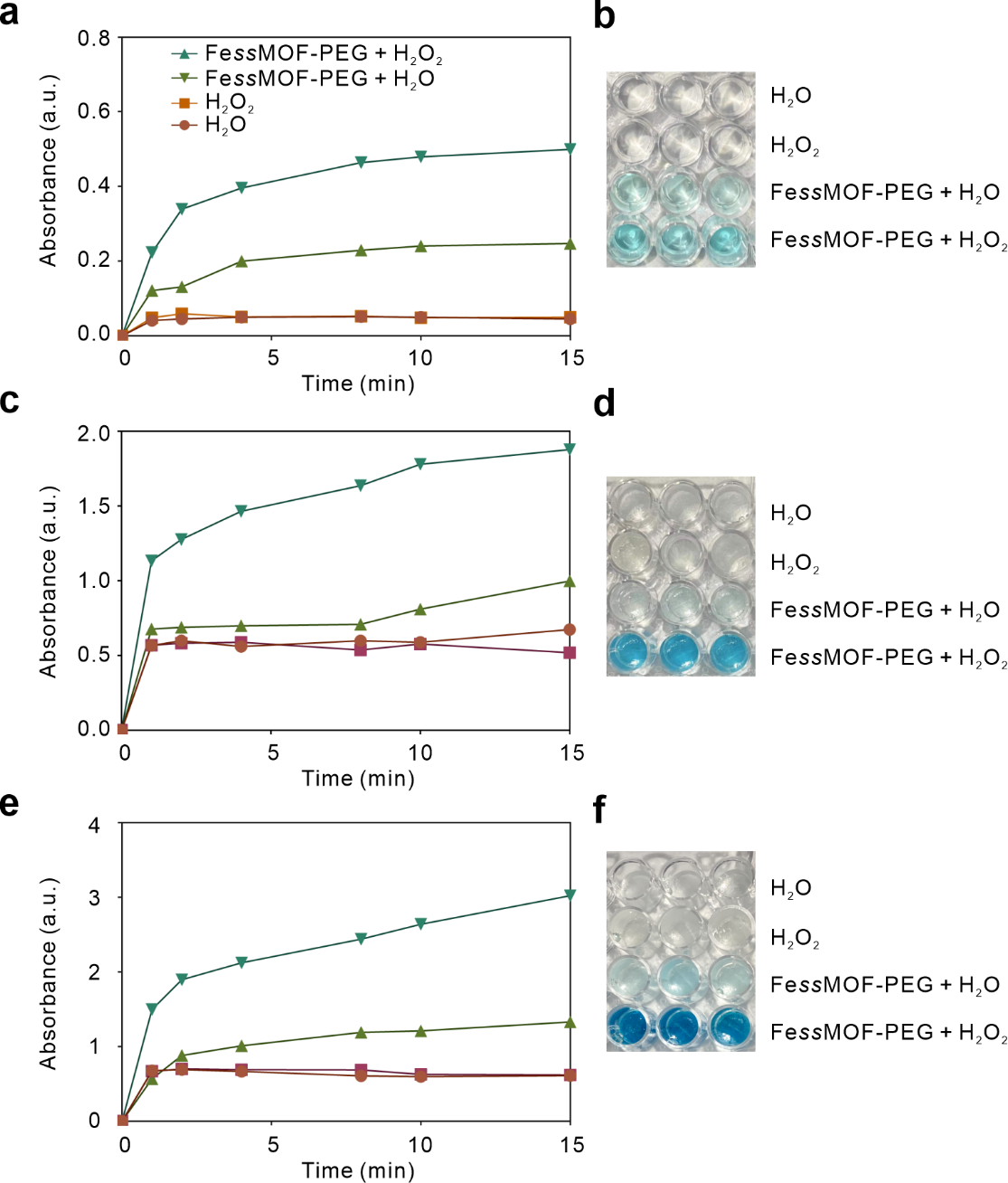


**Fig. S3**. The absorbance curves and images of FessMOF-PEG (50 μg/mL) and TMB at pH 7.4 (a, b), pH 6.0 (c, d), and pH 4.5 (e, f), with or without H_2_O_2_ treatment, from 0 to 15 minutes, respectively.

**
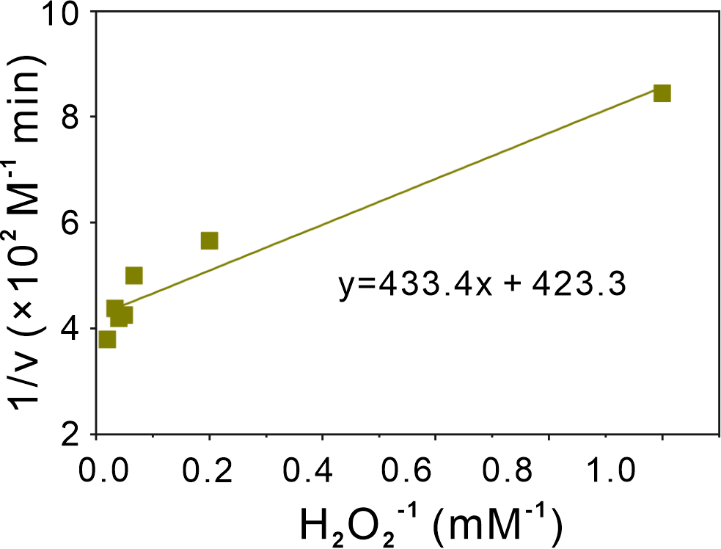
**

**Fig. S4**. Lineweaver-Burk plotting for Fe*ss*MOF-PEG with H_2_O_2_ as a substrate.

| Catalysts | Substances | K_m_ (mM) | V_max_ (M/s) | References |
| --- | --- | --- | --- | --- |
| FessMOF-PEG | H_2_O_2_ | 1.023 | 0.003.83 × 10^-8^ | This work |
| HRP | H_2_O_2_ | 3.7 | 8.71 × 10^-8^ | [1] |

**Table S1**. Comparison of K_m_ and V_max_ between FessMOF-PEG and HRP for H_2_O_2_.


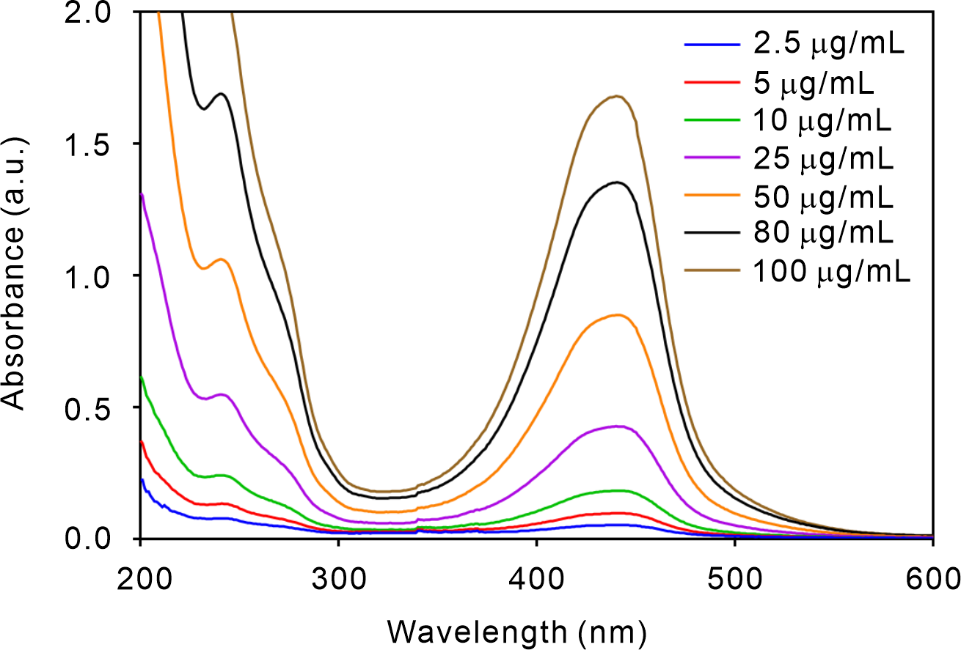


**Fig. S5**. UV-vis plots of different concentrations (2.5 - 100 μg/mL) of ActD.


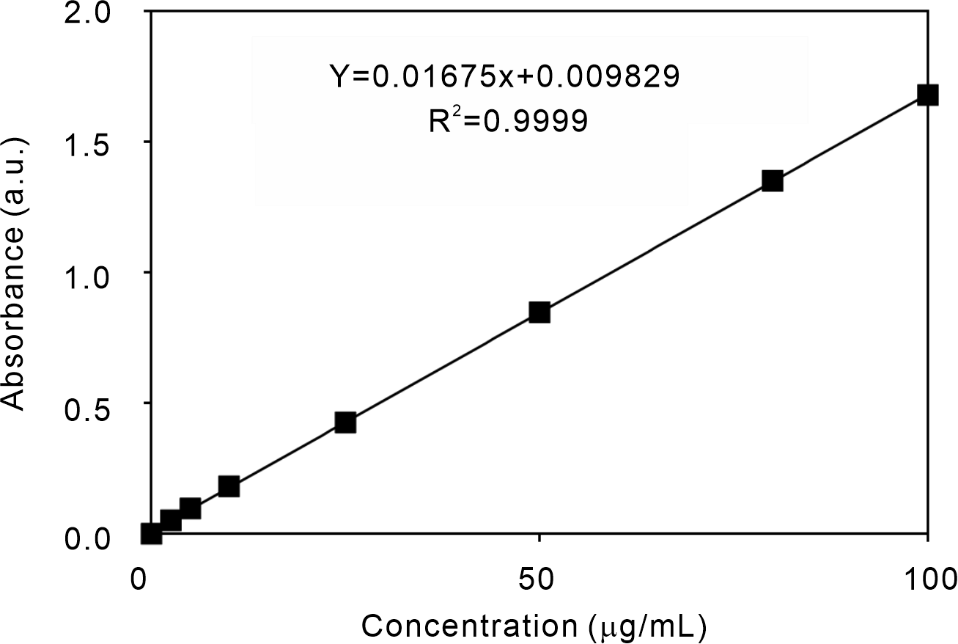


**Fig. S6**. Standard curve of ActD at 440 nm.


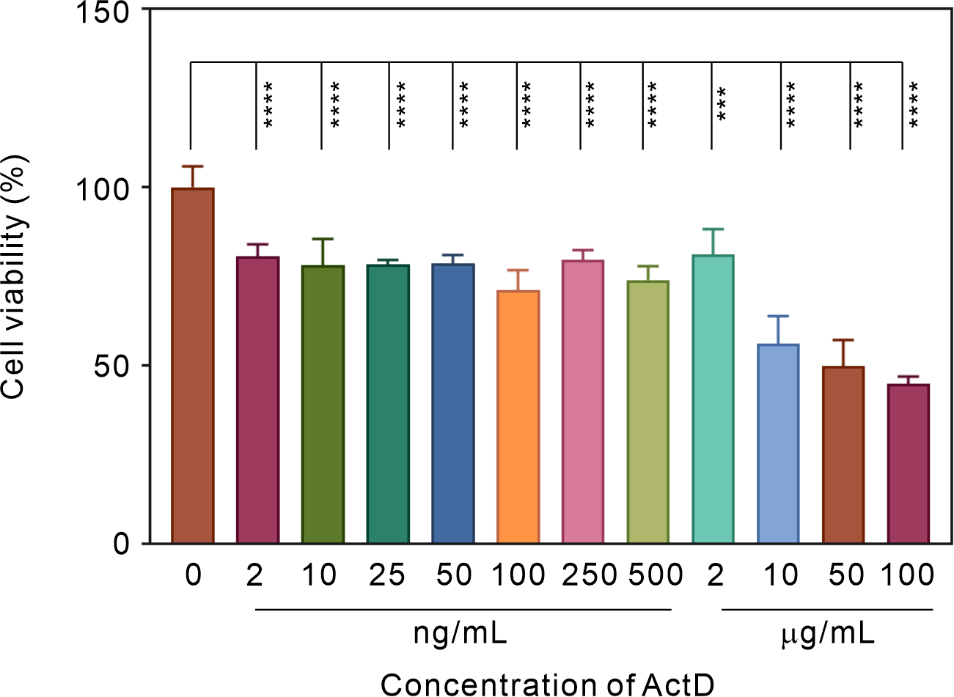


**Fig. S7**. Cell viability of 4T1 treated with different concentration of ActD for 12 hours. All data were presented as mean ± standard deviation. Statistical differences were calculated using two-tailed Student’s *t* test. Differences were considered significant when the p-value was less than or equal to 0.05. * p < 0.05, ** p < 0.01, *** p < 0.001, **** p < 0.0001, ns indicates no significance.


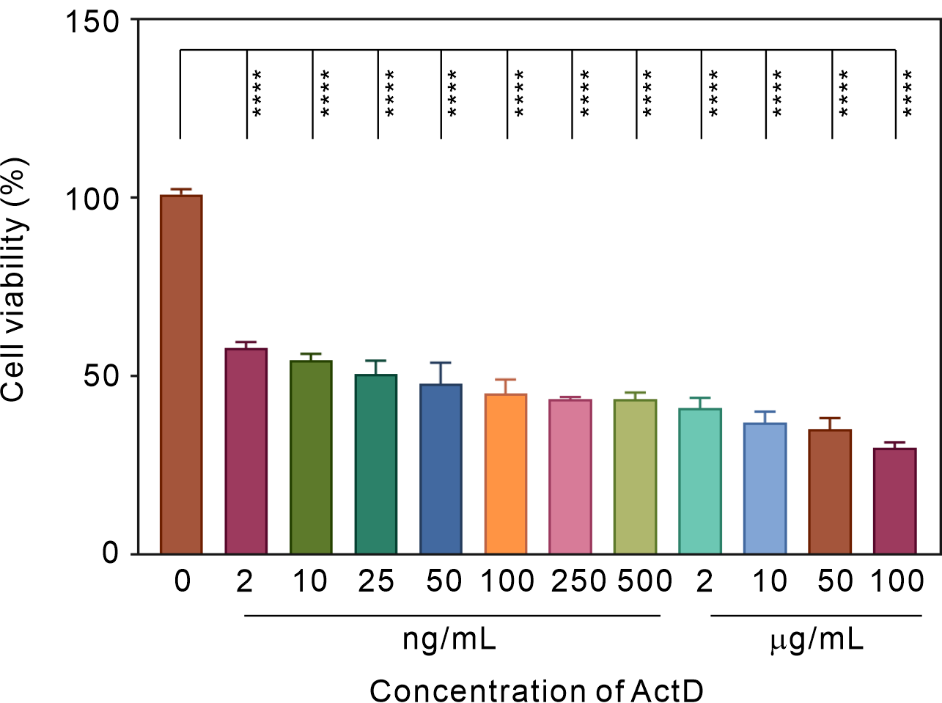


**Fig. S8**. Cell viability of 4T1 treated with different concentration of ActD for 24 hours. All data were presented as mean ± standard deviation. Statistical differences were calculated using two-tailed Student’s *t* test. Differences were considered significant when the p-value was less than or equal to 0.05. * p < 0.05, ** p < 0.01, *** p < 0.001, **** p < 0.0001, ns indicates no significance.


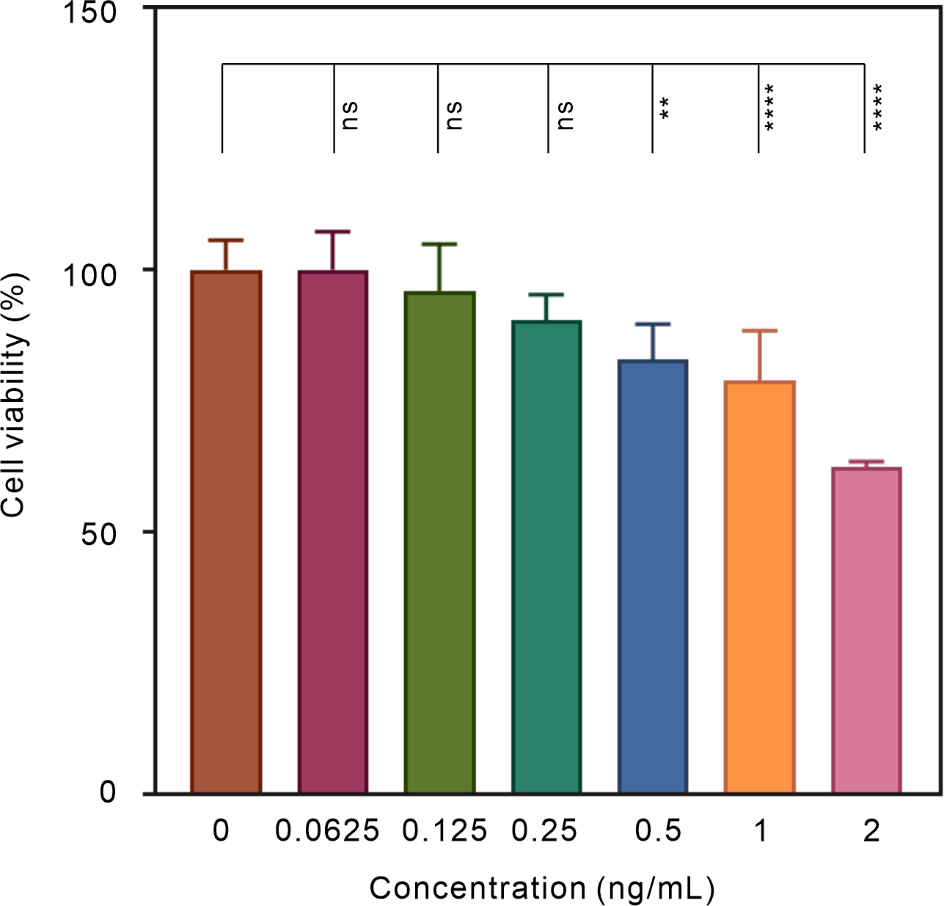


**Fig. S9**. Cell viability of 4T1 treated with different concentration of ActD for 24 hours. All data were presented as mean ± standard deviation. Statistical differences were calculated using two-tailed Student’s *t* test. Differences were considered significant when the p-value was less than or equal to 0.05. * p < 0.05, ** p < 0.01, *** p < 0.001, **** p < 0.0001, ns indicates no significance.


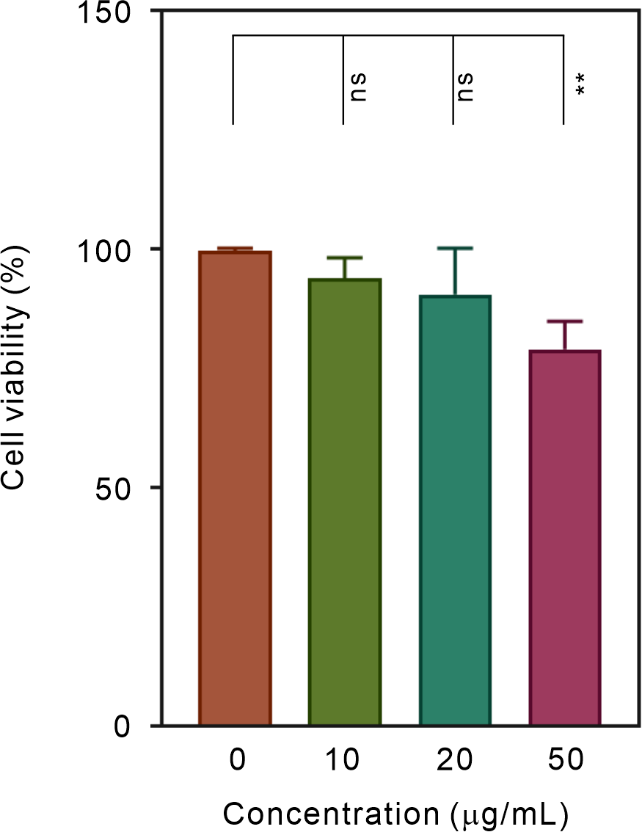


**Fig. S10**. Cell viability of 4T1 treated with different concentration of FessMOF-PEG for 24 hours. All data were presented as mean ± standard deviation. Statistical differences were calculated using two-tailed Student’s *t* test. Differences were considered significant when the p-value was less than or equal to 0.05. * p < 0.05, ** p < 0.01, *** p < 0.001, **** p < 0.0001, ns indicates no significance.

**
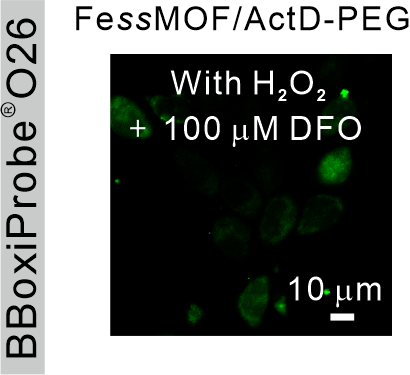
**

**Fig. S11**. ^•^OH staining by a BBoxiProbe®O26 fluorescent probe in 4T1 cells after different treatments.


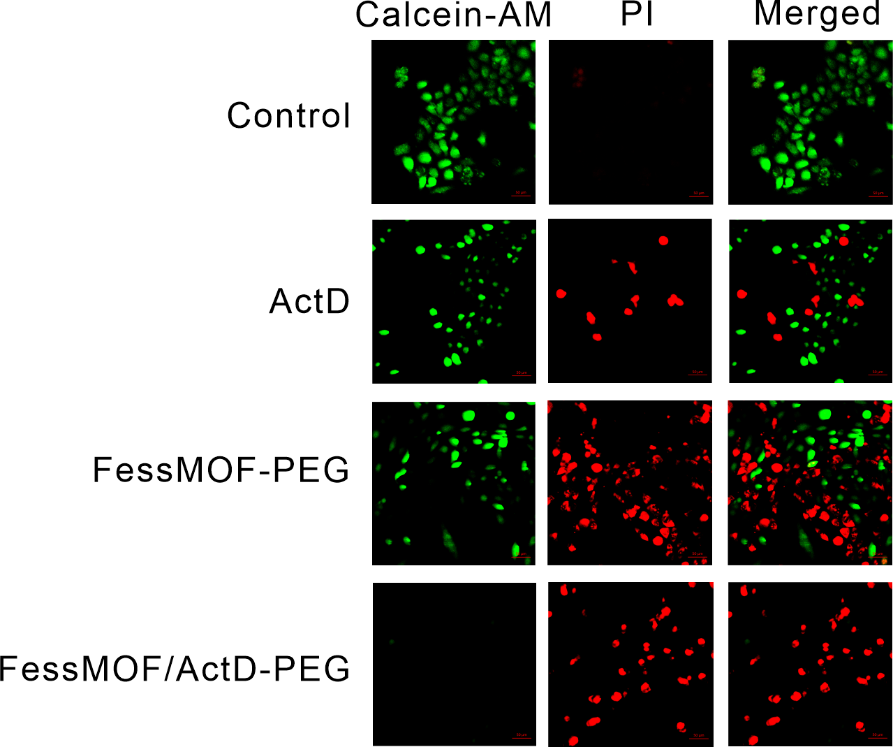


**Fig. S12**. Live/dead cell staining after various treatments.


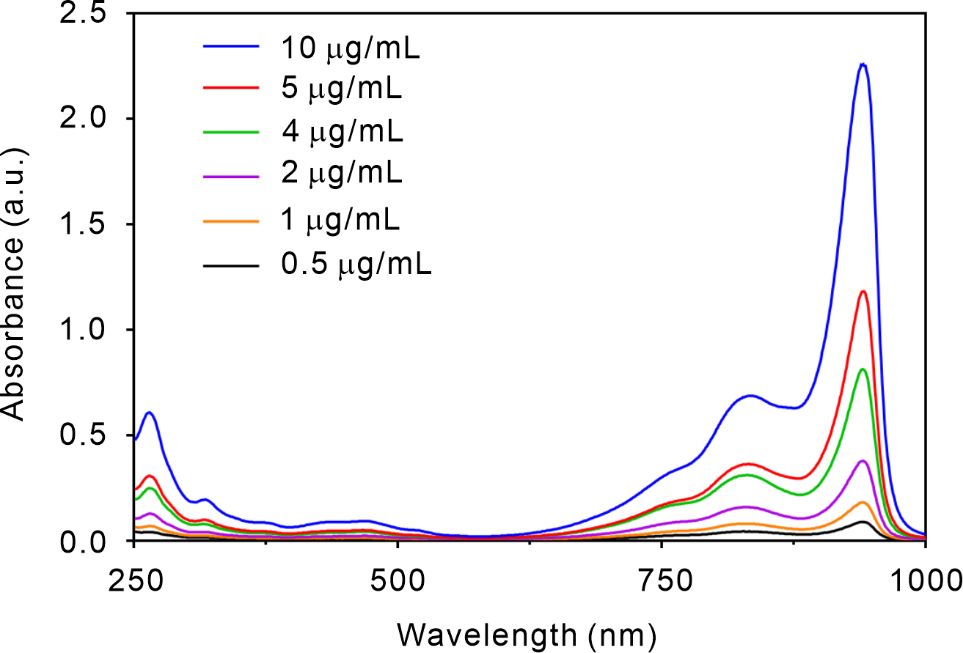


**Fig. S13**. UV-vis plots of different concentrations (0.5 - 10 μg/mL) of ICG.


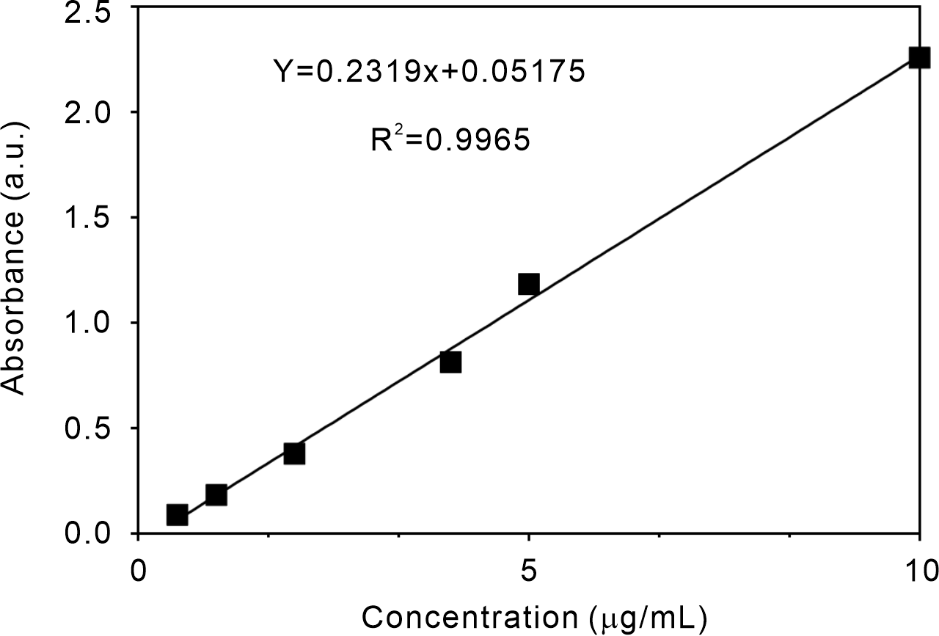


**Fig. S14**. Standard curve of ICG at 890 nm.


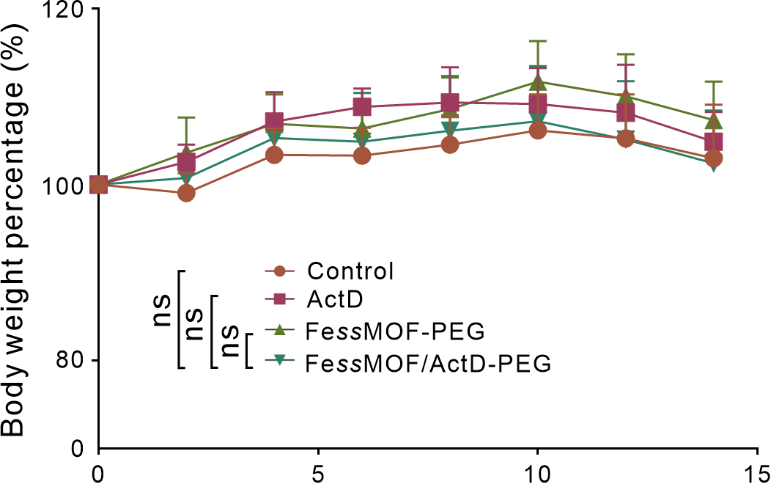


**Fig. S15**. The percentage of mice body weight.


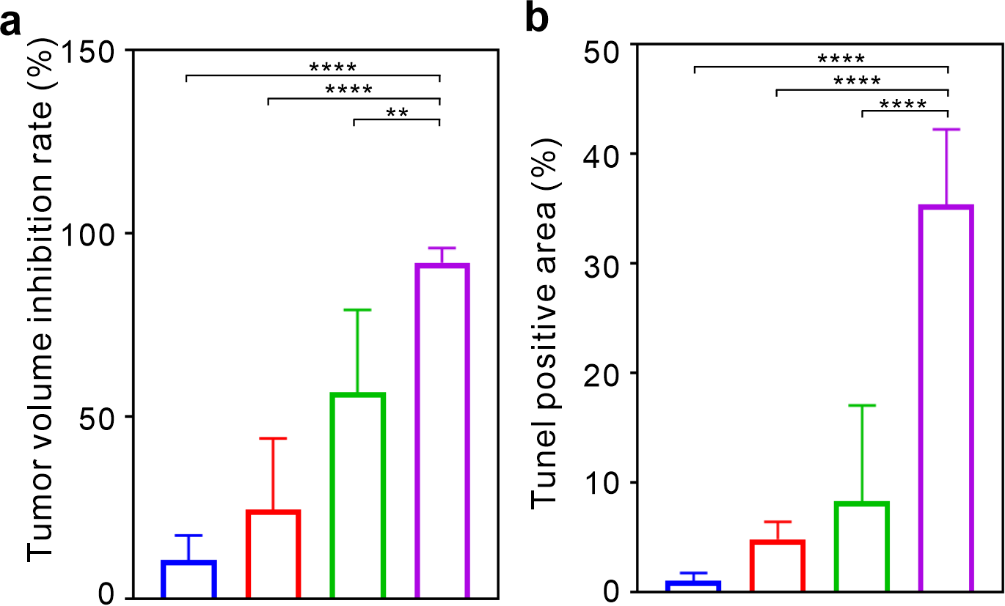
l

**Fig. S16**. (a) Inhibition rate of tumor volume. (b) TUNEL positive area of tumor. All data were presented as mean ± standard deviation. Statistical differences were calculated using two-tailed Student’s *t* test. Differences were considered significant when the p-value was less than or equal to 0.05. * p < 0.05, ** p < 0.01, *** p < 0.001, and **** p < 0.0001, ns indicates no significance.

**References**

1. Y. Huang, J. Ren, X. Qu, **Nanozymes: classification, catalytic mechanisms, activity regulation, and applications**, Chem. Rev. 119 (6) (2019) 4357-4412.
